# β-Blockers in the Environment: Challenges in Understanding Their Persistence and Ecological Impact

**DOI:** 10.3390/molecules30234630

**Published:** 2025-12-02

**Authors:** Anna Dzionek

**Affiliations:** Institute of Biology, Biotechnology and Environmental Protection, Faculty of Natural Sciences, University of Silesia in Katowice, Jagiellońska 28, 40-032 Katowice, Poland; anna.dzionek@us.edu.pl

**Keywords:** beta-blockers, emerging contaminants, pharmaceutical residues, biodegradation, ecotoxicology

## Abstract

β-blockers are among the most highly consumed cardiovascular drugs worldwide, resulting in their classification as persistent and bioactive pharmaceutical pollutants. This review provides a mechanistically oriented synthesis of their environmental release, stereochemical and matrix-dependent transformation, biotic and abiotic degradation, and the ecotoxicological significance of their degradation products. Wastewater treatment plants are identified as the primary, yet variably efficient, emission sources to aquatic systems. Molecular structure, chirality, and interactions with environmental matrices are highlighted as key factors influencing transformation behaviour and residue persistence. Current evidence indicates that β-blockers and several transformation products retain pharmacological activity, driving organism- and community-level effects at environmentally relevant exposures. Major limitations in the field stem from methodological heterogeneity and uneven regional and temporal coverage, which continue to weaken long-term risk evaluation. By unifying analytical chemistry, pharmacological mechanistics, and environmental toxicology evidence, this review advances the narrative from descriptive occurrence reports toward systematic evaluation of transformation product persistence, mechanism-dependent residue stability, and their ecological implications.

## 1. Introduction

Pharmaceuticals have been classified as major contaminants of emerging concern owing to their continuous release into the environment, biological activity at low concentrations, and incomplete removal in conventional wastewater treatment processes. Designed to elicit specific physiological effects in humans and animals, these compounds—including antibiotics, analgesics, antidepressants, and β-blockers—frequently enter aquatic ecosystems through municipal wastewater effluents, hospital discharges, and improper disposal practices [1,2]. Unlike many traditional pollutants, pharmaceuticals can persist in the environment, bioaccumulate, and cause sub-lethal or chronic effects on non-target organisms. Their detection in surface waters, sediments, and even drinking water has raised ecological and public health concerns. These concerns have intensified as evidence accumulates regarding their multi-generational and endocrine-disrupting impacts. Although pharmaceuticals are increasingly emphasized in environmental monitoring and regulations, many contaminants have yet to be incorporated into current management strategies. Thus, existing measures to prevent and mitigate risks continue to fall short [2].

β-adrenergic receptor blockers, commonly referred to as β-blockers, represent a pharmacologically diverse group of compounds that function as competitive antagonists at β-adrenergic receptors (β_1_, β_2_, and β_3_). These receptors are G protein-coupled receptors involved in regulating the cardiovascular, pulmonary, metabolic, and renal physiology within the sympathetic nervous system. Since their introduction in the 1960s, β-adrenergic blockers have played a critical role in reducing both morbidity and mortality associated with cardiovascular diseases. These agents are primarily used to manage hypertension, heart failure, tachycardia, cardiac arrhythmias, and coronary artery disease [3]. Currently, approximately 12 β-blockers are approved by the U.S. Food and Drug Administration. These include propranolol, metoprolol, atenolol, nadolol, timolol, acebutolol, betaxolol, bisoprolol, labetalol, nebivolol, carvedilol, and pindolol [4].

**Figure 1:**
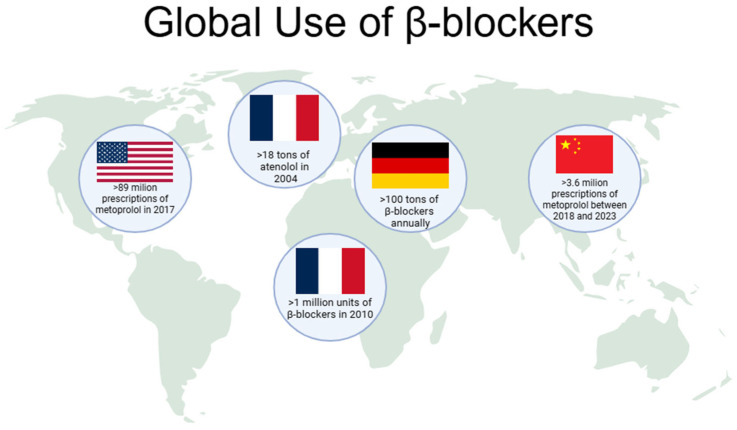
Global use of β-blockers [5,6].

β-blockers represent one of the most extensively prescribed classes of cardiovascular drugs worldwide (Figure 1). In France, consumption levels exceeded 18 tons of atenolol in 2004, while in Germany, the annual use of β-blockers has been estimated at approximately 100 tons per year. In the United States, β-blockers consistently rank among the top 100 most prescribed medications, with metoprolol alone accounting for more than 89 million prescriptions in 2017, making it the fourth most commonly dispensed drug nationally. Similarly, rapid growth has been observed in Asian markets: in China, metoprolol use has risen sharply, its annual consumption nearly doubling from about 27.9 kg in 2011 to 63.8 kg in 2015, in line with the increasing burden of hypertension. This upward trend continued, with over 3.6 million prescriptions issued between 2018 and 2023. In the United Arab Emirates, national pharmaceutical data indicate that β-blocker consumption surpassed one million units in 2010, further highlighting their expanding role in managing cardiovascular disease in the Middle East. Collectively, these statistics reflect the global dependence on β-blockers and the growing burden of cardiovascular disease that continues to drive their demand [5,6]. However, β-blockers’ global usage reflects wide economic disparity. In the year 2021–2022, the lowest absolute 30-day β-blocker prices were reported in Bangladesh and Pakistan, while the highest price ratios were in high-income North America and the U.S. Despite low absolute costs in some low- and middle-income countries, β-blocker-inclusive regimens were least affordable in low-income regions like Uganda due to catastrophic gross national income burden and reliance on unreliable public supply, limiting equitable β-blocker uptake [7].

Despite their structural and functional similarities, β-blocker subtypes vary in their ligand-binding preferences, cellular and subcellular distribution, interactions with signalling pathways, and regulatory processes. By blocking the binding of catecholamines (epinephrine and norepinephrine), β-blockers modulate downstream signalling pathways. In particular, they reduce cyclic AMP levels and inhibit protein kinase A-mediated phosphorylation, ultimately decreasing myocardial contractility, heart rate, and renin secretion [8,9].

β-blockers are classified based on their receptor selectivity, intrinsic sympathomimetic activity, and other pharmacodynamic properties. The most common system divides them into three generations. First-generation β-blockers, such as propranolol and nadolol, are non-selective agents that inhibit both β_1_- and β_2_-adrenergic receptors. This may induce bronchoconstriction, making them less suitable for patients with asthma or COPD. Second-generation β-blockers, including atenolol, metoprolol, and bisoprolol, are β_1_-selective (cardioselective) and preferentially inhibit receptors in cardiac tissue, with reduced effects on bronchial and vascular β_2_-receptors, especially at lower doses [10]. Third-generation β-blockers demonstrate variable β_1_-receptor specificity and possess additional vasodilatory properties. These arise from α_1_-adrenergic blockade (e.g., carvedilol, labetalol) or nitric oxide-mediated pathways (e.g., nebivolol) [3]. Another important classification parameter is intrinsic sympathomimetic activity, where some β-blockers (e.g., pindolol, acebutolol) partially activate β-receptors while simultaneously antagonizing them, resulting in less resting bradycardia [10].

**Figure 2:**
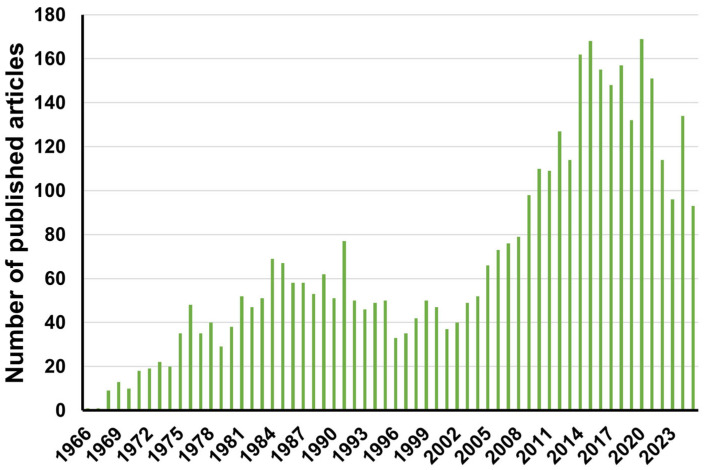
Annual publication trends on environmental fate and degradation products of β-blockers. Publication counts were obtained via a systematic literature search performed in PubMed using predefined keyword strings capturing both original research and review articles related to environmental fate, persistence, degradation pathways, transformation/degradation products, and analytical detection of β-blockers.

The literature trend reveals a strong long-term increase in peer-reviewed publications on β-blocker environmental fate, persistence, analytical detection, and transformation or degradation products from 1966 to 2024 (Figure 2). Early output (1966–1975) was low and gradually increased from approximately 15 articles/year. The first major rise occurred between 1976 and 1990, reaching an initial peak of 69 articles in the year 1984, aligned with expanded clinical adoption. A sharp acceleration began after 2008, with output rising from 98 to 168 articles/year, likely driven by advances in LC-MS/HPLC analysis and heightened concern over pharmaceutical persistence in aquatic systems. Although a slight decline is observed in recent years, the output remains high (93–114 articles/year), underscoring the continued relevance of degradation product research and the need for updated systematic synthesis.

Recent years show rapid expansion of environmental β-blocker research, reflecting improved analytical capabilities and increasing recognition of transformation product bioactivity. Current trajectories indicate a shift toward mechanism-resolved studies, including chiral inversion pathways, matrix-dependent biodegradation kinetics, and effect-relevant testing of degradation products. Future perspectives emphasize the need for standardized LC–MS screening protocols, inclusion of transformation residues in regulatory monitoring, and long-term multispecies effect evaluation, particularly in under-represented regions such as South Asia and low-income African catchments. Collectively, these trends highlight a field transitioning from detection inventories toward causal persistence frameworks and effect-anchored risk assessment.

Despite extensive reporting on β-blocker occurrence in surface waters and wastewater effluents, a critical gap remains in understanding their post-release behaviour [11]. In particular, the mechanisms driving their biodegradation, chiral/enantiomer-selective transformation, and interactions with environmental matrices are fragmented across disciplines, and evidence on the bioactivity and ecological impacts of metabolites and transformation products remains limited and inconsistent. This review focuses primarily on detection of environmental concentrations; it provides the first integrated evaluation linking (i) chemical–pharmacological properties, (ii) stereochemical fate, including enantioselective and chiral inversion pathways, (iii) matrix-mediated transformation processes, and (iv) ecotoxicological relevance of degradation products, assessed collectively rather than as isolated themes. By bridging analytical chemistry, pharmacology, and environmental toxicology, the review also highlights structure-dependent transformation mechanisms and identifies testable hypotheses explaining why bioactive β-blocker residues may persist despite treatment processes.

## 2. Occurrence and Distribution in the Environment

### 2.1. Main Emission Sources

β-blockers are frequently detected as environmental contaminants due to their extensive clinical use and incomplete removal during wastewater treatment. Additionally, the use of pharmaceuticals, including β-blockers, has risen significantly following the COVID-19 pandemic [12].

A primary pathway by which these compounds enter the environment is through municipal wastewater effluents. After administration, β-blockers are only partially metabolized in the human body, and both parent compounds and active metabolites are excreted via urine and feces [10]. These residues are transported to wastewater treatment plants (WWTPs), which are not specifically designed to eliminate pharmaceutical compounds [12]. Notably, propranolol, metoprolol, and atenolol are among the most frequently detected β-blockers in WWTP influents and effluents worldwide, with concentrations reaching 12.913 µg/L in the UK [13] and 5.249 µg/L in China [14]. Such concentrations are non-trivial, particularly given the chronic exposure risk for aquatic organisms.

As shown in Table 1, many β-blockers remain in effluents after treatment, even in technologically advanced regions. For example, in Germany and Spain—countries with stringent environmental regulations—measurable levels of β-blockers have been reported in both influent and effluent samples, reinforcing the global scope of the issue [15,16]. The temporal range of the data, covering more than a decade, reflects an ongoing trend: WWTP discharges act as a chronic source of β-blockers to aquatic environments, leading to their detection in rivers, lakes, and even groundwater systems. Although influent concentrations vary depending on local pharmaceutical consumption and population size, the consistent detection of β-blockers in effluents across different years and geographic regions (Europe, Asia, Africa) is evident (Table 1). Moreover, even less-studied β-blockers such as sotalol, betaxolol, and nadolol have been detected in WWTP influents, suggesting additional environmental release pathways that are not yet fully characterized.

In addition to municipal sources, hospital wastewater systems represent a distinct and often more concentrated pathway for the release of β-blockers and other pharmaceuticals. Hospitals and healthcare facilities generate wastewater containing a complex mixture of active pharmaceutical ingredients. β-blockers are commonly administered in high doses and frequencies, especially in intensive care, cardiology, and internal medicine departments. Unlike municipal wastewater, hospital effluents are typically untreated at the point of discharge and enter the public sewage system without prior pharmaceutical removal, contributing significantly to the overall pharmaceutical load entering WWTPs [12].

Atenolol and metoprolol exhibit the highest concentrations in hospital effluents. In Italy, atenolol has been reported to reach up to 5.8 µg/L, while metoprolol ranges between 0.74 and 1.1 µg/L [25]. Even higher levels have been observed in the USA, with atenolol at 3.79 µg/L and metoprolol at 3.54 µg/L [26]. These values exceed many concentrations reported in municipal WWTP influents (Table 1). Researchers suggest that in some urban areas, hospital effluents may account for a disproportionately large proportion of pharmaceutical residues, including β-blockers, despite making up a relatively small volume of total wastewater [25]. In China, metoprolol levels span a wide range (0.0078–10.002 µg/L), likely reflecting variability in prescription rates or discharge characteristics [14].

Propranolol also occurs consistently in hospital wastewater, with concentrations ranging from trace levels in Turkey (0.00097–0.0149 µg/L) to a peak of 0.366 µg/L in Southern Taiwan [12,27]. Although generally detected at lower levels than atenolol and metoprolol, propranolol remains a consistent contaminant, appearing across multiple continents and healthcare systems. Similarly, acebutolol, sotalol, pindolol, and timolol, although less frequently studied, have also been detected in measurable concentrations, further suggesting the diversity of β-blockers present in hospital effluents (Table 2).

Another major and often overlooked source of environmental contamination is the improper disposal of unused, expired, or surplus medications by individuals and institutions. Common practices such as flushing pharmaceuticals down toilets or sinks or discarding them into household trash result in direct environmental release or leaching from landfills into surface and groundwater. β-blockers, owing to their high stability and persistent activity, are particularly problematic, as their improper disposal circumvents waste treatment infrastructure entirely. Public awareness and regulatory enforcement of proper drug disposal remain limited in many regions, while pharmaceutical take-back programs, where available, are often underutilized [29]. However, successful initiatives in countries like Australia (e.g., NatRUM), New Zealand, and Austria demonstrate that, even without specific legislation, such programs can be effective and sustainable when supported by governmental or industry involvement and funding [30]. Improper disposal, therefore, not only contributes to ambient environmental contamination but also complicates monitoring efforts and risk assessments, particularly when multiple β-blockers and their metabolites co-occur in complex mixtures [29,31].

### 2.2. Environmental Presence in Aquatic Systems

Because of the combined emissions from municipal wastewater, hospital effluents, and improper disposal practices, β-blockers are now widely detected in aquatic environments, raising concerns about their persistence and ecological impact. Compounds such as atenolol, metoprolol, propranolol, and sotalol are frequently found in both surface and drinking waters, often at concentrations ranging from ng to µg/L (Table 3).

Propranolol appears in diverse aquatic environments, including rivers, lakes, and dam waters, with concentrations ranging from <ng/L to several tens of ng/L. The lowest reported mean values occurred in France (0.8–2 ng/L), while higher concentrations were observed in Poland’s Vistula River (1.2–38 ng/L) and particularly in the Llobregat River in Spain (54 ng/L) [32,35,36].

Atenolol is found more widely and at generally higher concentrations compared to propranolol, with exceptionally high levels reported in the Llobregat River (470 ng/L), the surface waters of South Africa (156–272 ng/L), and Uganda’s Lake Victoria (24–380 ng/L) [22,36,38].

Metoprolol exhibits some of the highest concentrations reported for β-blockers. Peak levels have been observed in the Vistula River (15–1190 ng/L) and in China’s Beiyun rivers (49.0–680.1 ng/L), with substantial levels also reported in Germany (102 ng/L) and the Llobregat River (90 ng/L) [33,35,36,41].

Other β-blockers, although less frequently monitored, are present in notable concentrations in specific surface waters. In the Vistula River, sotalol (34–1170 ng/L), bisoprolol (15–660 ng/L), and acebutolol (up to 270 ng/L) have all been detected at comparatively high levels. The Llobregat River similarly showed substantial contamination with sotalol (100 ng/L), bisoprolol (57 ng/L), acebutolol (44 ng/L), and labetalol (6 ng/L). These hotspots for multiple β-blockers highlight the lack of systematic monitoring in many other river systems, which prevents a comprehensive assessment of the true scale and distribution of such contamination.

To the best of our knowledge, the presence of β-blockers in tap water has not been studied frequently. Giebułtowicz et al. [35] examined the Vistula River, which is a source of water supply for Warsaw and its surroundings. The water was subjected to drinking-water treatments (ozonation, filtration with active carbon, slow filtration through biological filters, and chlorination) before being transferred to the tap water system. Unfortunately, these treatments were insufficient for pharmaceutical removal. Consequently, atenolol, metoprolol, and propranolol continued to be detected in Warsaw tap water at concentrations up to 15 ng/L.

The next level of environmental pollution is soil, where β-blockers from municipal and hospital wastewater discharges enter through wastewater irrigation, sludge application, and sediment deposition [42]. To date, only a few studies have specifically examined the occurrence and concentration levels of β-blockers in soil [14,43,44], resulting in a limited body of knowledge on their environmental distribution and behaviour within terrestrial ecosystems.

Soil sampling conducted along the Dagu Drainage Canal (Tianjin, China) revealed that metoprolol and sotalol were the most frequently detected β-blockers, whereas atenolol, propranolol, and nadolol were often absent or present below quantification limits. Topsoil concentrations of sotalol (102–258 ng/kg) and metoprolol (50–182 ng/kg) were consistently higher than subsoil concentrations, where sotalol ranged from not detected to 139 ng/kg and metoprolol from below quantification limits to 54 ng/kg. This vertical distribution was attributed to a combination of reduced contaminant infiltration into deeper layers and transformation processes occurring during downward migration. Overall, the β-blocker concentrations in soil were 2–4 orders of magnitude lower than those in sewage sludge from the adjacent canal [14].

Another study, conducted in Spain, reported metoprolol presence in soil samples. However, the measured concentrations did not exceed the established quantification limit [44]. In a different study, metoprolol was detected in sewage-irrigated Loess soil at concentrations ranging from 0.26 to 9.61 ng/kg [43].

The combined emissions from municipal wastewater, hospital effluents, and improper disposal practices form a multifaceted and persistent source of β-blockers in the environment. These emission pathways underline the urgent need for integrated environmental management strategies, including improved wastewater treatment technologies, more stringent hospital discharge regulations, and public education on safe pharmaceutical disposal. Only through coordinated action across healthcare, regulatory, and environmental sectors can the long-term risks associated with β-blocker contamination be effectively mitigated.

## 3. Physicochemical Properties and Environmental Behaviour

### 3.1. Chemical Structure and Chirality

Most β-blockers are derivatives of the isoprenaline scaffold. Their core consists of a substituted aromatic ring joined by an ether linkage to a propanolamine chain carrying a hydroxyl group and a secondary amino group. Structural modifications, such as substitutions on the aromatic moiety or alterations in the amine side chain, govern the compound’s lipophilicity, receptor selectivity, metabolic stability, and ability to penetrate the central nervous system [3].

Many β-blockers are chiral compounds, typically possessing one or more stereocenters, which results in the formation of enantiomers. These enantiomers differ in their interactions with biological targets, with the S-enantiomer generally responsible for the β-adrenergic blocking activity, whereas the R-enantiomer is often pharmacologically less active or inactive [45,46]. For example, in the human body, S-propranolol shows 1.5-fold higher bioavailability than the R-enantiomer, primarily due to stereoselective first-pass metabolism. Species-dependent differences also occur in the pharmacokinetics of enantioselective drugs. For instance, propranolol clearance demonstrates opposite stereoselectivity across species, with S > R in dogs and R > S in humans [47]. Most β-blockers are marketed as racemic mixtures, which is relevant not only in pharmacology but also in environmental behaviour assessment post-excretion [45,46].

### 3.2. Lipophilicity, Ionization, and Environmental Partitioning

The octanol-water partition coefficient (log K_ow_ or log P) is a fundamental parameter describing the lipophilicity of a compound. Elevated values are characteristic of hydrophobic substances, whereas lower values generally indicate higher aqueous solubility. Consequently, log K_ow_ is often used to infer environmental partitioning tendencies, including sorption to soils, sediments, and dissolved organic matter. β-blockers span a range of log K_ow_ values (Table 4), from lipophilic (e.g., propranolol) to hydrophilic (e.g., atenolol), informing both pharmacokinetics and environmental fate [48,49].

Most β-adrenergic blockers are weak bases (pK_a_ 8.0–9.7) that occur predominantly in their ionized form at neutral pH (Table 4). Because log K_ow_ values are derived from unionized species, they may not accurately predict real-world behaviour. In such cases, the distribution coefficient (log D), which integrates both ionized and unionized fractions at a given pH, provides a more accurate descriptor of solubility, membrane permeability, and partitioning in aqueous environments. Thus, compounds with similar log K_ow_ values may display markedly different aqueous solubilities and environmental mobility, reflecting the combined effects of ionization, hydrogen-bonding potential, and solid-state interactions [48,49]. For example, propranolol, a non-selective β-blocker, is highly lipophilic and readily crosses the blood–brain barrier, whereas atenolol is more hydrophilic and exhibits limited central nervous system penetration (Table 4) [49].

### 3.3. Sorption to Soils and Sediments

The environmental fate of β-blockers is largely influenced by their adsorption behaviour in soils and sediments, which determines their mobility, persistence, and bioavailability. Adsorption processes are governed by the physicochemical characteristics of the compounds (e.g., pK_a_, log K_ow_, and polarity) and the properties of the sorbent matrix (e.g., organic carbon content, cation exchange capacity, and mineral composition). Studies show that β-blockers exhibit moderate to strong adsorption to soils, particularly those rich in organic matter and clay minerals [50].

Early work by Kibbey et al. [54] demonstrated that hydrophobicity is a key predictor of β-blocker adsorption to mineral and alluvial materials, with propranolol possessing the highest log K_ow_ and exhibiting the strongest sorption affinity. Interestingly, electrostatic parameters such as zeta potential and pH relative to the mineral’s point of zero charge were poor predictors, despite β-blockers being predominantly cationic at environmental pH. However, the presence of anionic surfactants, frequently co-released in wastewater, substantially enhanced sorption, indicating that mixed-contaminant systems can alter adsorption dynamics and reduce pharmaceutical mobility.

Extending this understanding, Ramil et al. [50] examined nine β-blockers in aquatic-sediment systems and found detectable concentrations (up to 86 ng/g) in wastewater-impacted sediments. Sorption was linearly correlated with organic carbon content and particle size distribution. Complementary column studies by Schaffer et al. [55] identified cation exchange as the dominant sorption mechanism for atenolol and metoprolol in aquifer sediments, strongly regulated by calcium concentrations. Hydrophobic interactions played only a minor role, and conventional partitioning models based on log K_ow_ − K_oc_ correlations underestimated the retardation of cationic pharmaceuticals. These mechanistic insights highlight the importance of electrostatic interactions and site-specific mineralogy in predicting subsurface contaminant transport.

A more recent contribution by Smith et al. [56] examined the adsorption dynamics of propranolol and sotalol in soil and sediment matrices, both individually and in mixtures with each other and with copper (Cu^2+^). In this study, adsorption equilibrium was reached rapidly (<7 h). Moreover, propranolol exhibited significantly higher sorption affinity, with distribution coefficients (K_d_) of 82 L/kg and 51 L/kg in soil and sediment, respectively, compared to 5.2 L/kg and 0.7 L/kg for sotalol. This pronounced difference was attributed to the higher hydrophobicity and stronger cationic character of propranolol at environmental pH, which favours electrostatic and hydrophobic interactions with organic matter and negatively charged mineral surfaces. In contrast, the zwitterionic and partially anionic nature of sotalol limited its retention, resulting in greater aqueous mobility and potential bioavailability.

The influence of pH on sorption behaviour was notable. Sotalol adsorption decreased at higher pH values due to reduced cationic speciation and increased surface deprotonation. In contrast, propranolol adsorption remained relatively stable or slightly enhanced under alkaline conditions, likely reflecting increased neutral species and hydrophobic interactions. In binary mixtures, no significant competitive adsorption was observed; K_d_ values remained nearly unchanged, suggesting that adsorption sites were far from saturation and that the low sorption of sotalol did not hinder propranolol retention. Similarly, the presence of Cu^2+^ ions, despite their known potential for complexation with β-blockers, did not alter adsorption behaviour, implying negligible ternary surface complex formation or site competition under the tested conditions.

### 3.4. Chemical and Photochemical Stability

β-blockers exhibit varied chemical and photochemical stability profiles depending on their molecular structure and formulation. Many compounds, such as propranolol and atenolol, are relatively stable under neutral conditions but can undergo hydrolysis or oxidative degradation under extreme pH or elevated temperatures [57,58].

A comparative study by Krzek et al. [58] evaluated the degradation behaviour of atenolol, acebutolol, and propranolol in acidic environments, establishing a clear relationship between lipophilicity and chemical stability. Propranolol, the most lipophilic of the three, showed the greatest resistance to acid hydrolysis, whereas atenolol, with the lowest lipophilicity, degraded rapidly. This inverse relationship between the degradation rate constant and lipophilicity corresponded with increasing activation energy and half-lives from atenolol to propranolol, supporting the hypothesis that higher lipophilicity confers greater chemical robustness under acidic stress. Structural analyses using UV and NMR further confirmed the formation of degradation products via amide and ether hydrolysis, with propranolol showing minimal structural alteration.

Similarly, Peikova et al. [59] investigated the chemical stability of metoprolol tartrate under acidic (pH 2), neutral (pH 7.4), and alkaline (pH 9) conditions using a validated high-performance liquid chromatography method. Metoprolol showed pseudo-first-order degradation in acidic conditions, with a substantial decline in peak area and compound height over time, indicating notable acid sensitivity. However, it remained chemically stable at neutral and alkaline pH. This supports the broader conclusion that β-blockers, particularly those with higher lipophilicity, such as propranolol and metoprolol, exhibit greater resistance to chemical degradation.

The photostability of β-blockers is largely determined by their UV absorption spectra, which show that most compounds absorb only in the UV-C range (<280 nm) and are thus resistant to direct photolysis under natural sunlight. Only acebutolol, pindolol, propranolol, and timolol absorb above 280 nm and undergo partial direct photodegradation. In contrast, in sewage treatment plant effluents, degradation proceeds much faster (half-lives of 2–15 h) because of indirect photolysis mediated by hydroxyl radicals generated from nitrates and dissolved organic matter. Major transformation products include mono- and polyhydroxylated derivatives, indicating oxidation via radical addition rather than photodissociation [60,61].

Liu and Williams [62] comprehensively assessed the photodegradation behaviour of β-blockers in aquatic environments, investigating the direct photolysis kinetics of propranolol, atenolol, and metoprolol under simulated sunlight. Propranolol demonstrated the highest photoreactivity, with half-lives as short as 12–21 h under xenon lamp irradiation and estimated environmental half-lives ranging from <1 day in summer to approximately 12 days in winter at northern latitudes. In contrast, atenolol and metoprolol degraded more slowly, with estimated summer half-lives >2 weeks and winter half-lives extending to several months. These differences were attributed to structural features, particularly the naphthalene moiety in propranolol, which absorbs in the environmentally relevant UV-visible spectrum (295–800 nm) and promotes photoreactivity. Additionally, propranolol photolysis involved free radical intermediates and produced several identifiable degradation products, including aldehyde-containing isomers and deoxygenated structures.

In both natural waters and wastewaters, a wide range of inorganic ions and dissolved organic matter coexist, significantly influencing photochemical processes. Among these constituents, nitrate and dissolved organic matter, particularly fulvic acid derivatives, function as important photosensitizers in indirect photolysis. While undergoing photochemical reactions, nitrate generates hydroxyl radicals (·OH), which play a central role in initiating and propagating the degradation reactions of organic pollutants. The overall efficiency of photooxidation is strongly governed by environmental parameters such as nitrate concentration and pH. Specifically, increasing nitrate levels accelerates the photodegradation of atenolol, while the formation of ·OH through nitrate photolysis is itself pH dependent. Consequently, both nitrate abundance and pH determine the extent to which nitrate-driven radical pathways contribute to contaminant degradation in aquatic environments [60,63].

### 3.5. Bioaccumulation and Trophic Transfer

In the environment, lipophilic compounds penetrate biological membranes more readily, which facilitates their uptake by aquatic and terrestrial organisms. Their hydrophobicity is directly related to their bioaccumulation factor (BAF) and potential for trophic transfer in ecosystems. Compounds with log K_ow_ > 3.3 tend to bioaccumulate, while those with log K_ow_ > 5 may biomagnify through the food chain [64]. BAFs can also be estimated in situ by comparing the measured concentrations of lipophilic pharmaceuticals in fish tissues with their corresponding concentrations in ambient water. For β-blockers such as propranolol, this approach has revealed BAF values ranging from 1000 to 4000, classifying it as potentially bioaccumulative.

Notably, these field-derived BAFs were far higher than laboratory bioconcentration factors (36–160 in mussels), emphasizing that dietary uptake and fluctuating exposure strongly influence real-world accumulation. The inverse relationship observed between water concentrations and tissue BAFs indicates that propranolol may accumulate more efficiently at environmentally relevant, low concentrations, thereby enhancing its long-term risk potential [65].

The tissue-specific distribution of lipophilic β-blockers in *Hemiculter leucisculus* highlights the liver as the primary site of accumulation, reflecting its central role in detoxification, metabolism, and excretion. The markedly higher lipophilic pharmaceutical concentrations in the liver (6–10 times greater than those in muscle) suggest that hepatic cells not only biotransform but also store lipophilic compounds. This is concerning because the liver functions as a biochemical filter, and long-term accumulation may impair its physiological capacity while acting as a reservoir for internal redistribution of contaminants. Moreover, the observed presence of these compounds in the brain, although at lower concentrations, points to the ability of certain pharmaceuticals to cross the blood–brain barrier in non-target organisms.

Collectively, these findings indicate that while the liver is the dominant sink for lipophilic pharmaceuticals, the distribution to sensitive tissues such as the brain cannot be overlooked when assessing ecological risks [64,65].

Hydrophilic contaminants, in contrast, behave differently from lipophilic pollutants. Their high water solubility and low lipid partitioning facilitate persistence in aquatic environments, as they remain mobile and are not easily removed by sedimentation or sorption to organic matter. Moreover, these compounds often associate with proteins and phospholipid membranes, favouring accumulation in aqueous and protein-rich tissues such as blood, liver, and muscle, which may still give rise to chronic toxic effects despite their low lipid affinity. This is exemplified by β-blockers, whose molecular structures typically contain hydrogen bond-donating fragments such as hydroxyl and amine groups. These features enhance their polarity and enable extensive hydrogen bonding with water, proteins, and phospholipids, promoting their persistence in aquatic systems and their interaction with biological tissues through non-lipophilic pathways [66,67].

Recent studies demonstrate species-specific accumulation of atenolol, a hydrophilic β-blocker. The highest concentrations have been found in the piscivorous *S. brasiliensis*, intermediate levels in the omnivorous *P. maculatus* and *M. obtusidens*, and the lowest in the detritivorous *P. lineatus*. While this pattern may suggest a trophic-level effect, the evidence for biomagnification remains inconclusive and may instead reflect local exposure or species-specific metabolism [68]. Experimental studies in zebrafish further highlight tissue-specific accumulation of atenolol, with the liver showing the highest concentrations, followed by gut, gill, and brain. Bioconcentration factors ranged from 0.6 to 43 L/kg in the liver, where residual atenolol remained highest even after a 30-day depuration period. These results confirm that metabolically active organs play a key role in controlling internal atenolol distribution and clearance [67]. Supporting this, a marine food-web study in Hong Kong waters [69] demonstrated clear patterns of trophic dilution, with significantly negative slopes between atenolol concentrations and trophic level. This is consistent with the compound’s high hydrophilicity, rapid metabolism, and lower concentrations in higher-trophic species.

Collectively, these findings indicate that although atenolol can transiently accumulate in fish tissues, particularly in metabolically active organs, it generally undergoes trophic dilution rather than biomagnification in aquatic food webs.

## 4. Biological Degradation Mechanisms and Metabolites

The differential biodegradation behaviour of β-blockers under aerobic and anaerobic conditions carries significant implications for their environmental persistence and ecotoxicity. Because most river systems and lakes are oxygenated, aerobic degradation dominates, yet varying retention times and microbial activity result in site-specific persistence. Conversely, in anaerobic sediments or groundwater, β-blockers can remain stable for months, posing long-term exposure risks. In aerobic conditions, the biotransformation of β-blockers such as atenolol, metoprolol, and propranolol proceeds with variable efficiency depending on molecular structure and environmental context. During the wastewater treatment, β-blockers are primarily degraded through cometabolic pathways rather than direct microbial utilization, as their concentrations in wastewater are typically too low to serve as growth substrates. In contrast, anaerobic biodegradation of β-blockers is significantly slower and less efficient. The absence of oxygen limits the oxidative pathways commonly responsible for the initial transformation [70,71,72,73].

### 4.1. Atenolol

**Figure 3:**
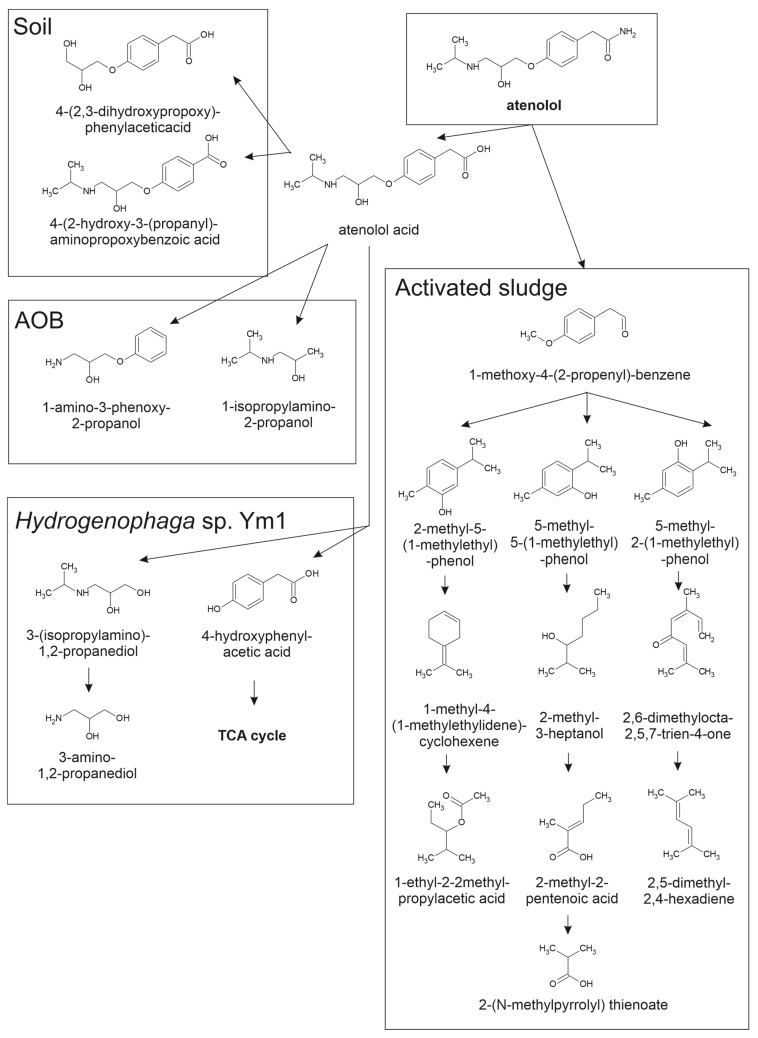
Overview of atenolol biotransformation or biodegradation pathways by various microorganisms [72,74,75,77].

Atenolol, a widely used and extensively studied β-blocker, undergoes significant aerobic biodegradation in both engineered treatment systems and natural environmental settings. Multiple studies have explored its microbial transformation pathways (Figure 3), identifying key enzymatic mechanisms and a diversity of intermediate products under oxic conditions [71,72,74,75,76,77].

In activated sludge systems, Xu et al. [74] demonstrated that atenolol degradation is enhanced by ammonium and ammonia-oxidizing bacteria (AOB). Under nitrifying conditions, 50% of atenolol (15 mg/L) was removed within 240 h, compared with only 40% in the absence of ammonium, supporting a cometabolic mechanism mediated by ammonia monooxygenase. Transformation products included atenolol acid, 1-isopropylamino-2-propanol, 1-amino-3-phenoxy-2-propanol, and an unidentified compound (P227), whereas only atenolol acid and P227 were detected under non-nitrifying conditions. The primary degradation route involved amide hydrolysis followed by ether bond cleavage and N-dealkylation, indicating the contributions of both hydrolytic and oxidative pathways. These findings highlight the influence of cometabolic AOB activity on β-blocker biodegradation during wastewater treatment.

Aerobic Sequencing Batch Reactors have demonstrated similarly high atenolol removal efficiencies under optimized conditions. Rezaei et al. [75] reported that, after an 80-day acclimation period, Sequencing Batch Reactors operated with activated sludge achieved up to 91% atenolol removal and 87% COD reduction at an influent concentration of 400 mg/L and a hydraulic retention time of 40 h. Atenolol served as both a carbon and energy source for microbial growth, with enzyme-mediated oxidation processes coupled to nitrification and denitrification. However, influent concentrations >500 mg/L caused microbial inhibition, sludge floc disintegration, and diminished treatment performance, underscoring the need for controlled loading rates. GC-MS analyses revealed phenolic and aliphatic intermediates, including benzene derivatives and pentenoic acids, driving a proposed degradation pathway that involves amino group cleavage, aromatic ring-opening oxidation, and eventual mineralization into linear organic acids.

Comparable biodegradation performance has been reported for bioactive sand filters used for drinking-water purification. Zhou et al. [76] observed up to 97.6% removal of atenolol (10 µg/L initial concentration) in quartz and manganese sand filters seeded with biofilms from full-scale treatment plants. Microbial biotransformation was the dominant removal mechanism, with atenolol amidohydrolase identified as the key enzyme catalyzing the conversion of atenolol to atenolol acid via amide hydrolysis. Metagenomic and transformation product analyses revealed that microbial consortia dominated by *Methylophilus*, *Methyloversatilis*, and *Nitrosomonas* mediated this pathway, suggesting a coupling of atenolol degradation with nitrogen cycling in the biofilter community.

Isolated pure cultures provide further mechanistic insight. Yi et al. [72] identified *Hydrogenophaga* sp. YM1 as a strain capable of efficient atenolol degradation under oxic conditions, achieving 82% removal of 300 µg/L within 72 h—significantly outperforming mixed cultures. The degradation pathway involved amide hydrolysis, ether bond cleavage, and aromatic ring oxidation, producing atenolol acid, 4-hydroxyphenylacetic acid, and small aliphatic compounds such as 3-(isopropylamino)-1,2-propanediol. Transcriptomic and RT-qPCR analyses showed that amidohydrolase and α-ketoglutarate-dependent dioxygenase (TfdA) are essential enzymes in this process, with complete mineralization proceeding via the homoprotocatechuate pathway through tricarboxylic acid cycle intermediates. Acetate supplementation generated α-ketoglutarate and reduced the equivalents necessary for dioxygenase function, thereby enhancing degradation by up to 37%. Notably, degradation efficiency decreased by over 40% under anoxic or anaerobic conditions, confirming the strict oxygen dependence of this pathway.

In natural environments, atenolol transforms primarily through biotic processes within soil–plant systems. Kodešová et al. [71] investigated atenolol dissipation in three agricultural soils—haplic chernozem, haplic cambisol, and arenosol—and observed rapid disappearance from soil and predominant accumulation in plant roots. Atenolol acid was identified as the major metabolite. Biotic degradation dominated the process, with minimal abiotic transformation. Plant species significantly affected degradation efficiency: lamb’s lettuce and spinach showed higher metabolic activity compared to radish and arugula, suggesting a strong influence of plant physiology on atenolol fate.

Koba et al. [77] expanded on this by evaluating atenolol degradation in 13 soils differing in texture and organic matter content. Atenolol transformed more rapidly than metoprolol, with degradation kinetics strongly correlated with soil structure and nutrient retention capacity. Amide hydrolysis to atenolol acid was the dominant pathway, with the metabolite accumulating transiently during early stages and undergoing subsequent oxidative mineralization. A secondary product (A1), characterized by low molecular weight and aromatic ring modifications, was also identified. Soils such as Chernozem and Cambisol supported faster degradation owing to higher microbial biomass and nutrient availability, while sandy and syenite-derived soils showed slower rates. After 40–60 days, atenolol (2 µg/g dry soil) and its metabolites were nearly eliminated, although persistence was greater in nutrient-poor matrices.

Together, these studies demonstrate that atenolol undergoes substantial aerobic degradation across diverse environments and microbial communities. While transformation is generally efficient in engineered systems and moderately effective in natural matrices, degradation pathways depend on oxygen availability, microbial consortia, and local environmental conditions. Amide hydrolysis to atenolol acid emerges as a common and critical step in most systems, typically followed by ether cleavage, aromatic ring oxidation, and eventual mineralization, although complete removal of transformation products may require system-specific optimization.

### 4.2. Metoprolol

**Figure 4:**
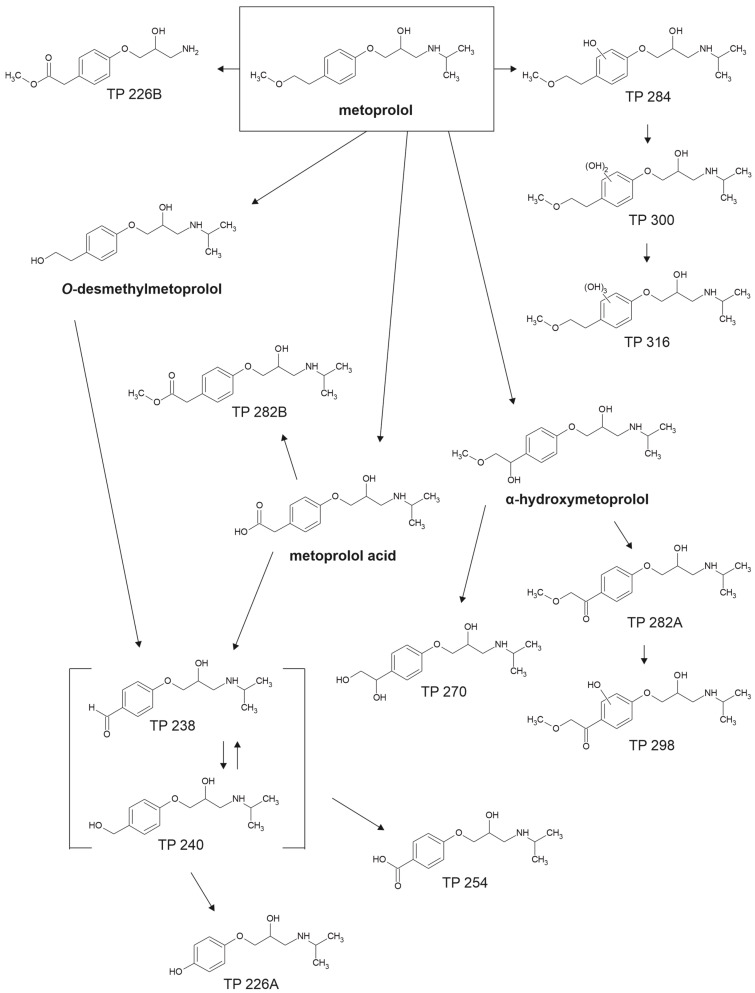
Biotransformation and biodegradation pathways of metoprolol [78]; modified.

Metoprolol, another commonly prescribed β-blocker, undergoes extensive biotransformation in diverse environments, including activated sludge, fungal treatment systems, and sediment microbial communities (Figure 4). Multiple studies have characterized its transformation pathways, key metabolites, and microbial actors under both oxic and anoxic conditions [70,78,79].

In activated sludge systems, Rubirola et al. [78] investigated the fate of metoprolol (1 mg/L) and reported up to 60% removal within 24 h and near elimination after 96 h under aerobic conditions. Adsorption and anaerobic degradation were minimal, confirming the dominant role of aerobic microbial processes. The transformation products included metoprolol acid (MTPA), α-hydroxymetoprolol (α-HMTP), and *O*-desmethylmetoprolol. Among these, MTPA emerged as the most persistent metabolite, accounting for approximately 40% of the parent compound after 96 h and remaining stable during extended treatment. In contrast, α-HMTP and *O*-desmethylmetoprolol were short-lived, peaking within 24 h before undergoing further transformation.

Complementing these findings, Jaén-Gil et al. [79] explored fungal-mediated metoprolol biodegradation as an emerging eco-friendly treatment strategy. Using the white-rot fungi *Ganoderma lucidum*, *Trametes versicolor*, and *Pleurotus ostreatus*, the study demonstrated oxidative transformation via cytochrome P450-dependent hydroxylation, *O*-dealkylation, and oxidation pathways. Fourteen distinct transformation products were detected, including α-HMTP, TP282A, and TP316, confirming broad enzymatic activity. *G. lucidum* achieved the highest removal efficiencies, approximately 51% for metoprolol and 77% for the recalcitrant MTPA within 15 days under aerobic conditions (2.0 ± 0.5 mg/L initial concentrations). However, persistent metabolites such as α-HMTP and TP240 remained in both the effluent and fungal biomass, suggesting incomplete mineralization and potential accumulation. Although fungal systems present promising alternatives, optimization is needed to mitigate residual metabolite persistence.

Beyond engineered systems, natural environments such as the hyporheic zone also facilitate metoprolol degradation under both oxic and anoxic conditions. Rutere et al. [70] examined metoprolol biotransformation in sediment microcosms, observing complete removal from the aqueous phase within 65 days under aerobic and 72 days under anaerobic conditions. Repeated metoprolol dosing accelerated degradation, indicating microbial adaptation and possible enzyme induction. Abiotic degradation was negligible, confirming the biological nature of the transformation. Under oxic conditions, MTPA was the dominant metabolite, whereas under anoxic conditions, both MTPA and α-HMTP appeared transiently, reflecting redox-dependent pathways.

Collectively, these findings demonstrate that metoprolol can be effectively biodegraded under diverse environmental conditions; however, the transformation routes and microbial consortia vary significantly with redox status and treatment system. Amide hydrolysis and oxidative transformations emerge as recurring mechanisms, yielding persistent intermediates such as metoprolol acid. While aerobic systems, both conventional and fungal, generally support more complete degradation, anaerobic environments also contribute, albeit with slower kinetics and differing microbial actors. The persistence of certain metabolites across systems underscores the need for advanced biotechnological strategies that not only target the parent compound removal but also that of the downstream, potentially recalcitrant metabolites.

### 4.3. Propranolol

Propranolol, a chiral β-blocker of high environmental relevance, exhibits slow and incomplete biodegradation in conventional wastewater treatment systems. Its removal is influenced by both its physicochemical properties, particularly its lipophilicity, and the metabolic capabilities of microbial communities under aerobic and anaerobic conditions [73,80,81,82].

Maurer et al. [80] reported average removal efficiencies in activated sludge systems, ranging from 35 to 50% at an initial concentration of 250 ng/L. Adsorption to biomass contributed <5% to total elimination, indicating that microbial oxidation was the dominant removal mechanism. The estimated pseudo-first-order biodegradation rate constant of 0.39 L/(d·g) COD reflected moderate microbial capacity for propranolol transformation in biological treatment environments. These findings highlight the challenges associated with removing lipophilic pharmaceuticals in conventional activated sludge systems.

**Figure 5:**
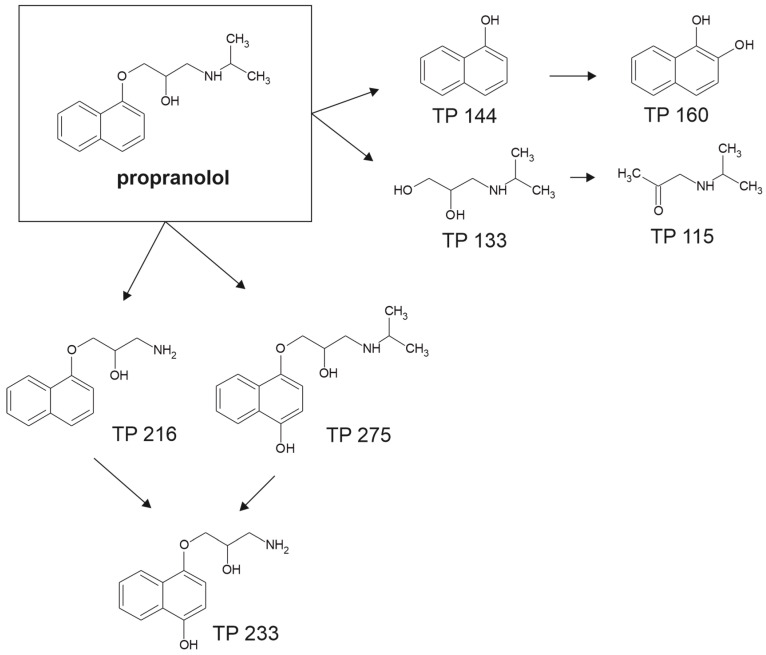
Biodegradation pathways of propranolol conducted by activated sludge inoculated with *Hydrogenophaga* sp. YM1 [78]; modified.

A more detailed mineralization pathway was provided by Yi et al. [73], who demonstrated complete aerobic degradation using activated sludge inoculated with *Hydrogenophaga* sp. YM1. The key enzymatic step involved *O*-dealkylation, catalyzed by TfdA. This reaction generated 1-naphthol and 1,2-dihydroxynaphthalene, which were converted to salicylate and fully mineralized via the tricarboxylic acid cycle (Figure 5). Molecular docking revealed that π-π stacking between propranolol’s naphthalene ring and Tyr73 in the TfdA active site reduced catalytic flexibility, limiting enzyme efficiency. However, co-feeding acetate improved degradation by stimulating NADH-dependent enzymatic systems and shifting the reaction kinetics from first-order to zero-order, indicating an energy-dependent transformation process.

Stereoselectivity also plays a significant role in propranolol’s environmental fate. Ribeiro et al. [81] demonstrated that biodegradation in activated sludge preferentially targets the S-enantiomer, which degrades 5–10% faster and has a shorter half-life than the R-enantiomer. This enantioselective pattern, which followed first-order kinetics, remained unaffected by acetate addition, confirming that it stems from intrinsic enzymatic stereospecificity rather than external substrate availability. Minimal abiotic transformation reinforced the biological origin of propranolol degradation. The shift in enantiomeric fraction toward the S-form suggests that these values can serve as reliable markers for assessing microbial degradation efficiency in treatment systems.

In contrast, propranolol removal in anaerobic environments is more complex and often limited. Tang et al. [82] evaluated its fate in Upflow Anaerobic Sludge Blanket reactors, where initial removal efficiencies were only around 20% at 100 mg/L but increased to approximately 60% with starch supplementation. This indicates that co-substrate availability and redox potential are critical factors influencing biotransformation under anoxic conditions. Microbial community analysis identified the involvement of *Thermotogae*, *Anaerolineae*, and *Bacteroidetes*, which contribute via co-metabolic pathways. Adsorption was minimal despite propranolol’s high hydrophobicity, as weak interactions with extracellular polymeric substances limited retention. Under oxidative stress, peroxidase enzymes catalyzed electron transfer reactions, enabling partial degradation. However, at elevated propranolol concentrations (400 mg/L), methanogenic activity was inhibited, leading to acetic acid accumulation and reduced methane yields, indicating metabolic disruption in anaerobic consortia.

Altogether, propranolol biodegradation is shaped by a combination of molecular, microbial, and environmental factors. While aerobic systems support moderate removal through biologically mediated *O*-dealkylation and stereoselective enzymatic oxidation, complete mineralization is rare without co-substrate stimulation. Anaerobic conditions allow only partial degradation via oxidative co-metabolism and are highly sensitive to propranolol concentration and carbon availability. Persistent intermediates, enantioselective degradation rates, and inhibition of key microbial functions underscore the need for treatment strategies tailored to the removal of chiral β-blockers such as propranolol.

## 5. Ecotoxicological Impact

Emerging research highlights significant ecotoxicological concerns associated with β-blockers and their transformation products in aquatic ecosystems. These pharmaceuticals exert pharmacological activity at nanomolar concentrations. Although they are typically detected at low ng/L levels in the environment, their ability to modulate adrenergic, serotonergic, and oxidative stress pathways in aquatic fauna presents a mechanism-driven risk that transcends simple concentration-based thresholds.

Although no universal safety threshold exists for β-blockers in surface waters, the literature-reported chronic Lowest Observed Effect Concentrations (LOECs) and sub-lethal effect ranges provide a basis for defining biologically meaningful exposure. Concentrations at or above 0.1–1 µg/L frequently overlap with receptor-mediated physiological disruption (e.g., cardiac modulation, metabolic stress, gill ion-regulation damage), while sensitive species exhibit behavioural and biochemical effects already within 10–100 ng/L. Therefore, wastewater detections in the high-ng/L to low-µg/L range should not be treated as insignificant when discharge is continuous and exposure chronic [33,83].

To contextualize biological significance, measured and reported β-blocker concentrations can be framed into ecological relevance brackets: low relevance (<10 ng/L), possible sub-lethal relevance (10–100 ng/L), population-influencing relevance (0.1–1 µg/L), and confirmed chronic effect overlap (>1 µg/L). In wastewater-impacted ecosystems, continuous discharge may sustain steady-state exposures within the latter two brackets, despite dilution below acute lethality levels [84,85,86,87,88,89,90].

### 5.1. Bacteria and Fungi

Ecotoxicological assessments using bacterial models such as *Vibrio fischeri* have reported moderate bioluminescence inhibition following exposure to propranolol and metoprolol. These responses are consistent with the drugs’ known membrane-targeting activities. However, *Arthrobacter globiformis*, a common soil bacterium, displayed reduced sensitivity to propranolol, likely due to strong sorption of the compound to the test substrate, which limited its bioavailability and subsequent toxic action. On the other hand, nadolol showed negligible toxicity in both bacterial models, emphasizing the importance of physicochemical properties and environmental matrices in shaping exposure dynamics and toxicity outcomes [91].

Fungal treatment processes show potential for degrading pharmaceutical pollutants, yet their ecotoxicological implications remain complex. Recent studies examining the fungal transformation of metoprolol and its primary metabolite, metoprolol acid, demonstrate that biotransformation does not necessarily equate to detoxification. Acute toxicity assays employing *V. fischeri* showed increases in toxicity of up to 29% following fungal treatment, with the magnitude depending on the fungal species involved. *G. lucidum*, in particular, showed the highest increase in toxicity for both metoprolol and metoprolol acid-treated samples. These outcomes contrast with those of conventional activated sludge treatments, which generally yield negligible changes in toxicity levels. While fungal degradation achieved substantial removal of parent compounds, it concurrently generated persistent and potentially more toxic transformation products. Notable among these were α-hydroxymetoprolol, TP238, and TP240, compounds detected across both aqueous and solid phases. Some transformation products exhibited preferential retention in fungal biomass, raising concerns regarding their environmental persistence and bioaccumulation. These findings underscore the critical need to include transformation products in ecotoxicological risk assessments, particularly in the context of advanced biological treatment technologies. Evaluating only parent compounds may significantly underestimate the ecological risks associated with pharmaceutical contaminants in aquatic systems [79].

### 5.2. Algae

A growing body of evidence indicates that β-blockers can disrupt fundamental physiological processes in algal species, even at sub-milligram concentrations. Exposure of the marine diatom *Phaeodactylum tricornutum* to propranolol (80–300 µg/L) resulted in pronounced photochemical and metabolic impairments, highlighting the susceptibility of primary producers to these pharmaceuticals. Despite lacking true β-adrenoceptors, the diatom possesses receptor-like kinases with functional similarity to animal receptors, which appear to mediate propranolol’s cellular effects.

Propranolol exposure inhibited photosystem II electron transport, leading to decreased light energy conversion efficiency and elevated oxidative stress. This was evidenced by increased catalase, superoxide dismutase, and lipid peroxidation activity. Moreover, the effects were accompanied by enhanced mitochondrial respiration and protein synthesis, reduced carbohydrate reserves, and increased lipid mobilization, indicating a shift from autotrophic to heterotrophic metabolism. Furthermore, the proportion of polyunsaturated fatty acids increased under propranolol stress, suggesting membrane restructuring as a compensatory response to oxidative conditions [92].

Studies on unicellular green algae have shown varying degrees of sensitivity. *Raphidocelis subcapitata*, when exposed for 72 h following OECD 201 guidelines, exhibited moderate growth inhibition in response to bisoprolol (median effective concentration [EC_50_] = 92.1 mg/L; chronic EC_10_ = 30.5 mg/L). In contrast, sotalol demonstrated negligible toxicity (EC_50_ values >100 mg/L). Although these concentrations are relatively high compared to environmental levels, they point toward the potential vulnerability of photosynthetic microorganisms to β-blocker exposure under realistic or cumulative exposure scenarios [89].

Similarly, the green algae *Scenedesmus vacuolatus* showed moderate sensitivity to propranolol (EC_50_ ≈ 24 mg/L), while metoprolol displayed reduced toxicity (EC_50_ = 75 mg/L), and nadolol remained ineffective up to 100 mg/L. The comparatively higher toxicity of propranolol may be attributed to its elevated lipophilicity, which enhances cellular uptake and increases the likelihood of interference with photosynthetic machinery [91].

### 5.3. Plants

The ecotoxicological effects of β-blockers on plants, particularly aquatic macrophytes, remain underexplored, with only limited data available. Among the few studies conducted, duckweed (*Lemna minor*) has been frequently used as a model organism because of its ecological relevance and sensitivity in aquatic environments.

Experimental exposure of *L. minor* to propranolol, metoprolol, and nadolol revealed relatively low phytotoxicity. Across tested concentrations (0.1–100 mg/L), none of the compounds produced significant changes in growth parameters or chlorophyll content. This limited sensitivity may reflect efficient detoxification mechanisms or restricted uptake through foliar tissues. However, given the relatively short exposure durations and elevated test concentrations in these studies, further investigation is required to fully understand the potential for chronic toxicity and bioaccumulation [91].

In another study following OECD Guideline 221, *L. minor* was exposed to bisoprolol and sotalol for 7 days, with growth assessed via frond number, frond area, and biomass. Bisoprolol exhibited EC_50_ values ranging from 313 to 345 mg/L, while sotalol showed EC_50_ > 1000 mg/L, confirming their low phytotoxic potential. Furthermore, chronic exposure to bisoprolol resulted in EC_10_ values of 115–136 mg/L, providing initial insight into sub-lethal effects [89].

### 5.4. Animals

Propranolol has demonstrated some of the strongest toxicological and physiological effects across aquatic taxa. Finn et al. [93] examined its impact (0.1–10 µg/L) on embryonic heart rate and development in *Oryzias latipes* (Japanese medaka) and *Danio rerio* (zebrafish). Both parental and embryonic exposures significantly reduced heart rate (bradycardia) at concentrations as low as 0.09 µg/L, with zebrafish embryos proving more sensitive. In zebrafish, propranolol also induced elongation of the heart tube and disrupted looping, whereas medaka embryos exhibited normal morphology despite the reduced heart rate. These results reveal the pronounced cardiac sensitivity of early vertebrate embryos to propranolol at near-environmental levels.

Margiotta-Casaluci et al. [94] confirmed the cardiovascular effects of propranolol in zebrafish larvae, aiming to evaluate their translational relevance for mammalian responses. Using 7-day-old larvae exposed for 1 h and 48 h under controlled laboratory conditions, they measured physiological parameters including heart rate, stroke volume, blood flow, and vessel diameter. Propranolol elicited a clear, concentration-dependent bradycardia (2–125 µM), with reductions in atrial and ventricular beat rates of up to 40% after short exposure. This was accompanied by a compensatory increase in stroke volume, reflecting a homeostatic cardiac adjustment. These effects closely mirrored those observed in mammals and humans treated with β-adrenergic antagonists, confirming the functional conservation of β-adrenergic signalling between zebrafish and higher vertebrates. Comparative meta-analysis showed that zebrafish responses aligned with mammalian data, including human data, in both direction and magnitude, supporting their suitability as a predictive vertebrate model for β-blocker pharmacodynamics and ecotoxicological assessment.

Propranolol also induced dose-dependent developmental, physiological, and behavioural effects in zebrafish [95]. Embryonic mortality increased (48 hpf LC_50_ = 21.6 mg/L), hatching was delayed at ≥20 mg/L, and altered heart rate responses to isoproterenol confirmed β-adrenergic disruption. Growth inhibition occurred at both low (0.0006 mg/L) and high (20 mg/L) concentrations, although reproduction remained unaffected. In adults, short-term exposure altered exploratory behaviour and induced biochemical changes, including elevated cholesterol and reduced sex hormone levels in males. This demonstrates that even sub-lethal propranolol doses can elicit biologically significant responses.

The marine polychaete *Hediste diversicolor* also responded strongly to propranolol contamination (0.05–5 ng/g of sediment) [84]. Exposure increased total lipid content, suggesting enhanced energy storage, although mitochondrial electron transport remained unaffected. Notably, propranolol reduced monoamine oxidase and cyclooxygenase activities, indicating neuroendocrine and anti-inflammatory interference analogous to vertebrate pharmacodynamics. These physiological disruptions corresponded with increased mortality at higher doses, highlighting the sensitivity of benthic invertebrates even at trace concentrations.

In the freshwater crustacean *Daphnia magna*, propranolol exhibited a receptor-mediated mode of action rather than nonspecific toxicity [85]. Exposure to concentrations of 0.3125–10 mg/L resulted in concentration-dependent bradycardia and reduced swimming activity after 6 h, suggesting cardiac-specific inhibition. Metabolomic analyses revealed changes in choline, lipid, amino acid, and glucose metabolism, consistent with reduced energy turnover. These results support the presence of conserved β-adrenergic signalling in *D. magna*, making it a sensitive model for receptor-specific pharmacological responses.

At the organismal level, chronic life-cycle exposure of *Pimephales promelas* (fathead minnow) to propranolol (0.87–8700 ng/L, 162–165 days) showed low acute toxicity but subtle reproductive and survival effects [86]. The highest dose (7800 ng/L) reduced juvenile survival by 15%. Interestingly, exposed fish showed enhanced reproductive output, with 70% higher egg production, larger clutch size, and decreased female gonadosomatic index, suggesting compensatory spawning behaviour. No developmental or reproductive impairments were detected in F1 offspring; however, these subtle life-history changes suggest population-level implications of chronic β-blocker exposure.

Triebskorn et al. [83] examined the effects of metoprolol in *Oncorhynchus mykiss* (rainbow trout) exposed for 28 days to concentrations of 1–100 µg/L. In the liver, metoprolol caused glycogen depletion, endoplasmic reticulum vesiculation, and increased macrophage activity, suggesting metabolic stress. Kidney effects were mild but included thickened basal membranes and increased macrophages, possibly due to altered blood pressure. In the gills, epithelial lifting, oedema, and hypertrophy of mucus and chloride cells indicated impaired ion regulation. Given a LOEC of 1 µg/L for fish hepatic and gill disruption wastewater detections, this concentration is not biologically negligible for species with conserved cardiac and neuroendocrine signalling, particularly under chronic exposure.

In the New Zealand mudsnail *Potamopyrgus antipodarum*, Feiner et al. [87] investigated the chronic effects of environmentally relevant sotalol concentrations (0.05–6.5 µg/L) over 56 days of exposure. Unexpectedly, low and medium concentrations (0.05 and 1.0 µg/L, respectively) caused a significant increase in embryo numbers, while the highest dose showed no effect, suggesting a hormetic response. Growth, offspring size, and mortality remained unaffected. These results show that sotalol can prolong reproductive activity and alter reproduction patterns in *P. antipodarum*, indicating that even low, sublethal levels of β-blockers may disrupt aquatic invertebrate populations and warrant further ecological risk assessment.

A mixture study by Salinas [88] demonstrated that combined exposure to propranolol and metoprolol induced considerable physiological and histological alterations in *Crassostrea virginica* (American oyster) after 1 week of exposure. At both low and high environmentally relevant doses (low: 50 ng/L propranolol + 250 ng/L metoprolol; high: 250 ng/L propranolol + 650 ng/L metoprolol), oysters exhibited gill and digestive gland damage, reduced mucous secretion, and increased haemocyte density, indicating immune activation. Extrapallial fluid analysis showed reductions in pH and glucose, while biochemical assays revealed increased nitrosative stress (3-nitrotyrosine protein expression) and decreased acetylcholinesterase activity. These findings suggest that β-blocker mixtures can impair metabolic, neural, and immune functions in oysters.

In contrast, broader ecotoxicological screenings indicate relatively low sensitivity of many aquatic invertebrates and fish to bisoprolol and sotalol [89]. In *Daphnia similis*, bisoprolol caused acute immobilization (EC_50_ = 93.1 mg/L) and reduced reproduction in chronic exposure (EC_10_ = 3.6 mg/L), while sotalol showed minimal effects (EC_50_ > 300 mg/L). The cnidarian *Hydra attenuata* exhibited moderate sensitivity to bisoprolol (EC_50_ = 115–193 mg/L; EC_10_ = 43 mg/L). In contrast, *D. rerio* embryos and larvae showed no mortality, developmental abnormalities, or behavioural changes up to 1000 mg/L, except for minor heart rate and locomotion effects at unrealistically high concentrations.

In the freshwater rotifer *Brachionus calyciflorus*, parent propranolol exhibited substantial toxicity, whereas its photodegraded mixtures were significantly less harmful, confirming the detoxifying effect of solar-driven transformation. In reproduction inhibition assays, propranolol caused complete mortality at 10 mg/L and reduced offspring production by >70% at 3.2 mg/L. Conversely, phototransformed samples produced no observable effects at comparable concentrations following 24–48 h of irradiation. The inability to derive EC_50_ values for the photodegraded mixtures further underscored their diminished biological potency. These findings indicate that the parent β-blocker exerts its toxicity through direct physiological interference, likely via β-adrenergic receptor interactions and membrane stabilization, while the oxidized, polar transformation products lack such activity. Thus, photolytic degradation of propranolol substantially alleviates sublethal stress and reproductive impairment in rotifer populations, demonstrating a key natural attenuation pathway for β-blockers in aquatic ecosystems [90].

### 5.5. Humans

Despite the growing concern regarding the environmental persistence and transformation of β-blockers, there is a notable lack of studies assessing their toxic effects on human systems. In this context, in vitro testing using human cell lines represents a crucial bridge between environmental and biomedical toxicology, allowing evaluation of both environmental relevance and human biological sensitivity under controlled conditions [96,97].

A pivotal early investigation by Cheong et al. [96] systematically assessed the in vitro cytotoxicity of eight clinically used β-blockers: propranolol, alprenolol, atenolol, labetalol, metoprolol, pindolol, timolol, and bisoprolol, using six human-derived cell lines, including ocular (corneal and retinal epithelial) and dermal (keratinocyte and fibroblast) models. Using the MTT assay, the authors demonstrated that cytotoxicity occurred rapidly (within 30 min) and was highly compound-dependent, differing by nearly 60-fold across the tested drugs. The most cytotoxic compound was propranolol (IC_50_ ≈ 0.4 mM), whereas atenolol was the least toxic (IC_50_ ≈ 23–35 mM), with timolol, a widely used ophthalmic agent, showing moderate toxicity (IC_50_ ≈ 7 mM). Interestingly, cytotoxicity was consistent across all cell types, suggesting a non–cell-specific, physicochemical mechanism likely related to lipophilicity rather than receptor-mediated effects. These findings provided early evidence that human ocular cells are reliable surrogates for toxicity assessment and that immortalized human lines can effectively replace animal models in biocompatibility testing of ophthalmic drugs.

Building on this foundation, later research extended the focus from parent compounds to their environmental transformation products, which often display unexpected biological activity. Četojević-Simin et al. [97] examined the cytotoxicity of metoprolol and its photocatalytic degradation mixtures formed using TiO_2_-based catalysts (Wackherr and Degussa P25) in two human cell lines: HT-29 (colon adenocarcinoma) and MRC-5 (fetal lung fibroblasts). The study revealed that while the parent compound exhibited limited toxicity, its degradation products, especially those formed using Degussa P25, significantly reduced cell viability in a time-dependent manner. The highest cytotoxicity occurred after 8–24 h of photocatalysis, correlating with the accumulation of hydroxylated and quinone-like intermediates. Human-derived cell lines demonstrated measurable sensitivity, with HT-29 cells showing moderate inhibition and MRC-5 fibroblasts displaying low but significant cytotoxicity at longer exposures. These results highlighted that incomplete degradation of β-blockers during water treatment can paradoxically increase biological toxicity, emphasizing the need for complete mineralization in advanced oxidation processes.

Together, these two studies illustrate the evolution of β-blocker toxicity research, from assessing the inherent cytotoxicity of parent compounds on human ocular tissues to evaluating the environmental and biological hazards of their transformation products. Collectively, they underscore the value of human cell-based in vitro systems as a powerful and ethically sound alternative for environmental toxicology, capable of detecting subtle but relevant effects of pharmaceuticals and their degradation products on human health.

### 5.6. Microcosms Community-Level Effects

Microcosm experiments provide valuable insights into community-level and indirect ecological effects of β-blockers, which are often overlooked in single-species assays. Exposure to propranolol (0.1 and 1 mg/L) within Baltic Sea microcosms has been shown to induce a complex set of both direct and indirect ecological responses, mediated through species interactions and trophic linkages. The most sensitive organism, *Mytilus trossulus* (blue mussel), exhibited elevated respiration, increased mortality, and high bioconcentration of propranolol, consistent with previous single-species toxicity findings. However, interactions with multiple taxa, such as macroalgae (*Ceramium tenuicorne*) and amphipods (*Gammarus* spp.), modified these outcomes through compensatory ecological feedback. Mussel deterioration enriched the water with ammonium and total nitrogen, triggering a trophic cascade in which amphipods shifted feeding from algae to stressed mussels. This shift reduced algal grazing and enhanced algal carbon content and apparent growth. These community-level buffering processes attenuated the direct toxic effects observed in simpler test systems, resulting in smaller or even positive responses in some taxa. Thus, propranolol exposure altered ecosystem structure and function through both physiological stress and indirect nutrient-mediated pathways. These findings demonstrate that microcosm complexity is critical for predicting the ecological consequences of β-blocker contamination in low-biodiversity coastal environments such as the Baltic Sea [98].

In a separate study using hyporheic zone microcosms, metoprolol exposure under redox-delineated conditions revealed selective effects on microbial functional groups. Community analyses based on 16S rRNA and rRNA gene sequencing showed that metoprolol altered bacterial activity rather than diversity, affecting key taxa involved in nitrogen and carbon cycling. Under oxic conditions, Sphingomonadaceae (notably *Novosphingobium* spp.) were stimulated, suggesting a role in aerobic *O*-demethylation. Conversely, nitrifying taxa such as *Nitrospiraceae* and *Nitrosomonadaceae* were inhibited, implying potential disruption of nitrification. In anoxic microcosms, Enterobacteriaceae (e.g., *Escherichia*/*Shigella* spp.) and Promicromonosporaceae (*Cellulosimicrobium* spp.) were enriched, consistent with anaerobic *O*-demethylation and hydroxylation processes, whereas nitrifiers and Anaerolineaceae declined. These findings demonstrate that metoprolol can modulate microbial function and biogeochemical processes even at environmentally relevant concentrations and that the hyporheic zone microbiome contributes significantly to its natural attenuation through redox-dependent biodegradation pathways [70].

## 6. Knowledge Gaps and Research Challenges

Although β-blockers are among the most frequently detected pharmaceutical residues in aquatic and terrestrial environments [33,99], major uncertainties remain regarding their environmental behaviour, transformation, and ecological effects. Current knowledge is fragmented, with significant research gaps limiting our ability to perform comprehensive risk assessments and design effective mitigation strategies.

A key challenge involves the monitoring of β-blockers in natural water bodies. Diverse analytical approaches, sample preparation protocols, and detection limits undermine data comparability across regions. Standardized analytical procedures and inter-laboratory validation are needed to generate reliable global datasets [1]. Monitoring efforts also remain geographically and temporally limited—many rivers, lakes, and coastal areas have never been surveyed. Broader, more frequent monitoring campaigns are necessary, and the resulting data should be accessible through open, harmonized databases. A global platform compiling concentration data, analytical methods, and environmental metadata would enhance transparency, support meta-analyses, and facilitate international collaboration. It is important to emphasize that the apparent scarcity of recent concentration data for β-blockers does not indicate a limitation of this review but rather reflects a broader global monitoring gap. Although the consumption of β-blockers continues to rise, these compounds are still not routinely included in most water-quality surveillance programs. Currently, no international regulatory thresholds or standardized freshwater monitoring frameworks exist for β-blocker discharges or environmental concentrations. Consequently, the detections reported here represent an emerging contaminant concern and underscore the urgent need to establish normative guidelines and systematic monitoring programs, an issue expected to be addressed in forthcoming EU wastewater directives. Moreover, the number of peer-reviewed studies reporting quantitative levels of β-blockers in wastewater treatment plant effluents, hospital discharges, surface waters, or drinking water remains limited. As a result, reliable assessment of temporal trends is not yet possible, highlighting the critical need for more frequent and standardized monitoring to better capture current environmental conditions and emerging contamination patterns.

Geographic and environmental bias further constrain current research. Most studies have been conducted in Europe [32,35,37,99], North America [40], and East Asia [33,34], whereas data from the Global South remain scarce. Differences in climate, wastewater infrastructure, and pharmaceutical usage patterns suggest that β-blocker dynamics may differ substantially across regions. Additionally, research has largely focused on aquatic environments [33,34,35,37,38,39,40,99], while soils, sediments, and groundwater systems—important sinks and potential secondary sources—remain understudied. Investigations in these compartments are particularly rare, despite the increasing use of treated wastewater and biosolids in agriculture, which may facilitate their accumulation and long-term release.

Although Table 1 summarizes β-blocker concentrations in WWTP influent and effluent samples from various regions, direct comparison of treatment efficiencies across studies remains challenging. Wastewater treatment processes typically involve multiple, interdependent steps, and overall removal performance often reflects the cumulative effect of several treatment stages rather than a single technology. Biological treatment, in particular, plays a decisive role, yet its efficacy depends strongly on microbial community composition, which varies regionally and temporally. Consequently, it is not possible to generalize that one technology consistently outperforms others under all conditions. Moreover, reported removal rates frequently overlook the formation and persistence of transformation products. In many cases, apparent degradation reflects only partial modification of the parent compound (e.g., hydroxylation) [78], which may not correspond to true detoxification or mineralization. Additional factors such as seasonal variability, influent composition, and the presence of co-contaminants further obscure cross-study comparisons. Future research should therefore aim to evaluate β-blocker removal within integrated treatment frameworks, combining physical, chemical, and biological processes, while systematically monitoring transformation products and microbial community dynamics to identify mechanistically effective and environmentally sustainable solutions.

Microbiological and enzymatic aspects of β-blocker degradation also remain poorly understood. Existing studies typically describe overall biodegradation kinetics [50,70,77,81] but provide little insight into the specific microbial taxa, metabolic pathways, or enzymes involved. Such information is crucial for elucidating the environmental fate of β-blockers, particularly under variable redox and nutrient conditions. Advanced molecular techniques, including metagenomics, metatranscriptomics, and enzyme assays, remain underutilized. Strengthening our understanding of microbial adaptation, co-metabolic degradation, and enantioselective transformation potential would greatly improve predictive models.

The broader environmental transformation processes, beyond biodegradation, also involve abiotic mechanisms, such as photodegradation and interactions with porous or catalytic materials or advanced oxidation processes. These pathways, while not the main focus of this review, represent an important complementary perspective on β-blocker fate in aquatic systems. Studies have demonstrated that photochemical reactions and heterogeneous catalysis can significantly influence compound persistence, yet their interplay with microbial degradation and environmental conditions remains poorly understood [62,100]. Moreover, laboratory and field investigations reveal that co-contaminants, natural organic matter, and light exposure can modify both biodegradation rates and photostability [63,70]. Bridging the knowledge gap between biotic and abiotic processes is therefore essential for accurately assessing the overall removal efficiency, transformation pathways, and ecological risks associated with β-blockers in real environmental matrices.

The issue of chirality adds further complications. Many β-blockers are chiral and exist as enantiomeric pairs that can differ significantly in pharmacological potency, biodegradability, and toxicity [45,46]. However, most environmental monitoring and degradation studies treat them as racemic mixtures, overlooking enantioselective transformations that may lead to enantiomeric enrichment and altered toxicity or persistence profiles [81]. Therefore, developing enantioselective analytical methods and incorporating stereochemistry into risk assessments are essential steps forward.

Ecotoxicological research on β-blockers also remains constrained by methodological and conceptual gaps. Most toxicity studies examine only a few model organisms and focus on acute endpoints [85,89,91,92,93,95], providing an incomplete picture of long-term and community-level effects. Chronic low-dose exposures, which are more representative of real environmental conditions [87], can produce subtle yet ecologically significant changes such as altered microbial functionality, endocrine disruption, and shifts in primary productivity. Furthermore, studies addressing mixture toxicity are scarce, despite the frequent co-occurrence of multiple β-blockers and other pharmaceuticals in aquatic systems [35]. Additive, synergistic, or antagonistic interactions complicate the identification of single-compound effects and call for more realistic mixture toxicity experiments.

One of the least understood aspects of β-blocker fate in the environment concerns the toxicity of their transformation by-products. Although degradation pathways have been described for several compounds, the biological consequences of these reactions remain poorly characterized. Evidence to date suggests that transformation does not always equate to detoxification—fungal and photocatalytic processes, in particular, may generate intermediates that are more toxic than the parent drug. Without systematic ecotoxicological testing, it remains difficult to determine whether current treatment strategies genuinely mitigate risk or merely shift toxicity to less recognizable forms. Bridging this gap requires integrated chemical and toxicological studies capable of linking transformation pathways to ecological outcomes.

Taken together, these limitations underscore the need for a more holistic ecotoxicological framework. Such an approach should integrate molecular-level transformations and microbial processes with population- and community-scale ecological outcomes. Incorporating both direct toxicity endpoints and indirect ecosystem feedback, particularly under chronic low-dose exposures, would enable more realistic environmental risk assessments. Future research must prioritize combined evaluations of bioaccumulation potential and functional disruptions across trophic levels. Addressing these gaps will not only refine our understanding of the environmental fate of β-blockers but also guide the development of sustainable management strategies for pharmaceutical pollution.

## 7. Conclusions

Current knowledge on the environmental fate and effects of β-blockers shows both substantial progress and persistent gaps. These compounds are now well-documented in aquatic environments, with emerging work elucidating their physicochemical behaviour, degradation pathways, and ecotoxicological profiles. However, inconsistent monitoring, limited understanding of microbial and enantioselective transformations, and a geographic bias in available data continue to constrain effective risk assessment. Future research must focus on the development of standardized, globally comparable monitoring protocols, mechanistic studies on microbial degradation under environmentally relevant conditions, and evaluations of mixture toxicity and chronic exposure. These directions align with regulatory needs for more comprehensive environmental safety assessments and may serve as strategic goals for research funding and policy development. Improved understanding of β-blocker persistence is essential for designing informed wastewater treatment upgrades, formulating evidence-based water quality standards, and integrating pharmaceutical pollution into broader environmental management frameworks. Addressing these challenges will support the development of more resilient and ecologically sustainable water and sanitation policies amid increasing pharmaceutical loads.

## Figures and Tables

**Table 1:** Occurrence of selected β-blockers in WWTP influent and effluent.

β-Blocker	Country/Region	Mean Concentration in the WWTP Influent [µg/L]	Mean Concentration in the WWTP Effluent [µg/L]	Year of Samples Collection	Reference
Propranolol	China, Tianjin	0.180	Information not provided	2013	[14]
	Turkey	0.00331–0.00743	0.00064	Information not provided	[12]
	Australia	0.018–0.151	0.036–0.076	2012–2013	[17]
	USA, New York	0.377–2.000	0.299–0.852	2013	[18]
	Germany	0.064	0.062	2008	[15]
	Romania	0.0268	0.0194	2018	[19]
	Portugal	0.320	-	2017	[20]
	Spain	0.123	0.073	2014–2015	[16]
	UK	0.557	0.265	2007	[13]
Metoprolol	China, Tianjin	0.692–3.5	0.522–5.249	2013	[14]
	Turkey	0.00743–0.0868	0.0397	Information not provided	[12]
	Germany	2.0	2.0	2008	[15]
	UK	0.075	0.069	2007	[13]
Atenolol	China	0.00554	Information not provided	2016–2021	[21]
	Turkey	0.154–0.424	0.138–0.163	Information not provided	[12]
	Australia	0.255–0.300	0.075–0.135	2012–2013	[17]
	USA, New York	0.003–0.097	0.023–0.153	2013	[18]
	China, Tianjin	1.283	0.0487	2013	[14]
	Germany	1.3	0.4	2008	[15]
	Romania	0.2086	0.0946	2018	[19]
	Spain	1.620	1.140	2014–2015	[16]
	UK	12.913	2.870	2007	[13]
	South Africa	1.593–2.541	0.364–0.712	2015	[22]
	Argentina	Information not provided	0.2–1.7	Information not provided	[23]
	Tunisia	2.198–0.388	1.244–0.420	2014	[24]
Nadolol	China	0.00001	Information not provided	2016–2021	[21]
Sotalol	China	0.00454	Information not provided	2016–2021	[21]
	China, Tianjin	0.243	Information not provided	2013	[14]
	Turkey	0.00743–0.081	0.0304	Information not provided	[12]
	Germany	2.1	1.9	2008	[15]
Betaxolol	Romania	0.0449	0.0169	2018	[19]
Bisoprolol	Germany	0.48	0.31	2008	[15]
	Romania	0.1001	0.051	2018	[19]

**Table 2:** Detected concentrations of selected β-blockers in hospital wastewaters in the literature.

β-Blocker	Country/Region	Mean Concentration [µg/L]	Year of Samples Collection	Reference
Propranolol	Southern Taiwan	0.3660	2009	[27]
	Italy	0.023–0.085	2009–2010	[26]
	China, Tianjin	0.110–0.158	2013	[14]
	Turkey	0.00097–0.0149	Information not provided	[12]
	USA	0.01–0.200	2013	[26]
Atenolol	Southern Taiwan	0.0941	2009	[27]
	Italy	2.4–5.8	2009–2010	[25]
	Saudi Arabia	0.329–0.730	2014	[28]
	China, Tianjin	0.0009–0.0036	2013	[14]
	Turkey	0.0353–0.156	Information not provided	[12]
	USA	1.370–3.790	2013	[26]
Metoprolol	Southern Taiwan	0.5813	2009	[27]
	Italy	0.74–1.1	2009–2010	[25]
	China, Tianjin	0.0078–10.002	2013	[14]
	Turkey	0.00669–0.0182	Information not provided	[12]
	USA	0.750–3.540	2013	[26]
Acebutolol	Southern Taiwan	0.0975	2009	[27]
Nadolol	Italy	0.0012	2009–2010	[25]
Pindolol	Italy	0.038–0.12	2009–2010	[25]
Sotalol	Italy	0.048–5.1	2009–2010	[25]
	Turkey	0.00041–0.00754	Information not provided	[12]
	USA	0.1–0.53	2013	[26]
Timolol	Italy	0.033	2009–2010	[25]
Betaxolol	Italy	0.01–0.011	2009–2010	[25]

**Table 3:** Global occurrence of β-blockers in surface waters and tap water.

β-Blocker	Country/Region	Water Body	Mean Concentration [ng/L]	Year of Samples Collection	Reference
Propranolol	France	surface waters (urban and rural dam of water, river, and lakes)	0.8–2	2007–2008	[32]
	China	Beiyun rivers	0–5.86	2016	[33]
	China	Surface waters from 31 provinces	0.25	2014–2015	[34]
	Poland	Vistula River	1.2–38	2013–2014	[35]
	Poland	Tap water (Warsaw)	7.0	2013–2014	[35]
	Spain	Llobregat River	54	2008–2009	[36]
	Hungary	Streams near Budapest	36	2010–2011	[37]
Atenolol	South Africa	River	156–272	2015	[22]
	Uganda	Lake Victoria	24–380	2018	[38]
	Brasil	Paranoá Lake	34.7–90	2017	[39]
	France	Surface waters (urban and rural dam of water, river and lakes)	0.2–34	2007–2008	[32]
	China	Beiyun rivers	1.4–6.2	2016	[33]
	Poland	Vistula River	1.4–104	2013–2014	[35]
	Poland	Tap water (Warsaw)	1.5	2013–2014	[35]
	Mexico	Apatlaco River	4–32	2015–2016	[40]
	Spain	Llobregat River	470	2008–2009	[36]
	Germany	Surface waters	8.8	2012	[41]
	Hungary	Streams near Budapest	55	2010–2011	[37]
Metoprolol	France	Surface waters (urban and rural dam of water, river and lakes)	0.5–2	2007–2008	[32]
	Uganda	Lake Victoria	0.4–21	2018	[38]
	China	Beiyun rivers	49.0–680.1	2016	[33]
	China	Surface waters from 31 provinces	5.4	2014–2015	[34]
	Poland	Vistula River	15–1190	2013–2014	[35]
	Poland	Tap water (Warsaw)	14	2013–2014	[35]
	Spain	Llobregat River	90	2008–2009	[36]
	Germany	Surface waters	102	2012	[41]
	Hungary	Streams near Budapest	1230	2010–2011	[37]
Sotalol	Poland	Vistula River	34–1170	2013–2014	[35]
	Poland	Tap water (Warsaw)	16	2013–2014	[35]
	Spain	Llobregat River	100	2008–2009	[36]
	Germany	Surface waters	25	2012	[41]
	Hungary	Streams near Budapest	160	2010–2011	[37]
Bisoprolol	Poland	Vistula River	15–660	2013–2014	[35]
	Poland	Tap water (Warsaw)	8.5	2013–2014	[35]
	Spain	Llobregat River	57	2008–2009	[36]
Labetalol	Poland	Vistula River	1.9	2013–2014	[35]
	Spain	Llobregat River	6	2008–2009	[36]
Acebutolol	Poland	Vistula River	2.5–270	2013–2014	[35]
	Poland	Tap water (Warsaw)	1.2	2013–2014	[35]
	Spain	Llobregat River	44	2008–2009	[36]
	Hungary	Streams near Budapest	98	2010–2011	[37]
Carvedilol	Hungary	Streams near Budapest	41	2010–2011	[37]

**Table 4:** Structures and physicochemical properties of the selected β-blockers [49,50,51,52,53].

Compound (CAS Number)	Structure	pKa	log K_ow_	log D in pH 7.4	Solubility in Water at 25 °C (mg/L)
Propranolol (525-66-6)	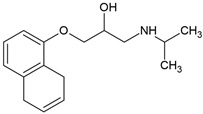	9.5	3.5	1.29	61.7
Metoprolol (51384-51-1)	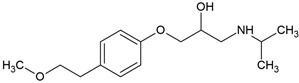	9.7	1.9	−0.28	16,900
Atenolol (29122-68-7)	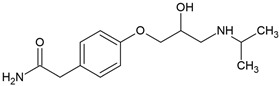	9.6	0.16	−1.61	13,300
Nadolol (42200-33-9)	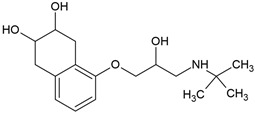	9.7	0.8	−1.30	8330
Timolol (26839-75-8)	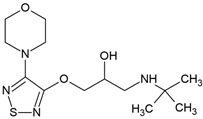	9.2	1.76	−0.52	2470
Acebutolol (37517-30-9)	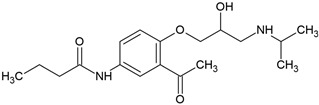	9.2	1.71	3.4	259
Betaxolol (63659-18-7)	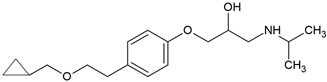	9.4	3.26	0.42	451
Bisoprolol (66722-44-9)	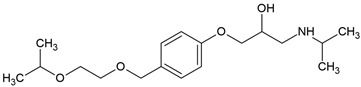	9.6	2.15	−0.02	2240
Labetalol (36894-69-6)	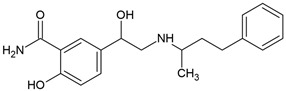	9.3	2.6	1.09	117
Carvedilol (72956-09-3)	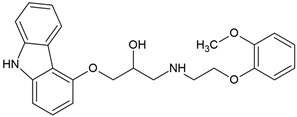	8.0	4.19	3.5	insoluble
Pindolol (13523-86-9)	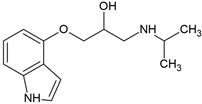	9.7	1.75	−0.10	insoluble
Sotalol (3930-20-9)	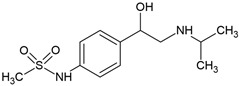	8.2	0.2	−1.50	5510

## Data Availability

No new data were created or analyzed in this study. Data sharing is not applicable to this article.

## References

[1] Ratnasari A., Thakur S.S., Zainiyah I.F., Boopathy R., Wikurendra E.A. (2025). An Overarching Critical Review on Beta-Blocker Biodegradation: Occurrence, Ecotoxicity, and Their Pathways in Water Environments. Curr. Pollut. Rep..

[2] Feng W., Deng Y., Yang F., Miao Q., Ngien S.K. (2023). Systematic Review of Contaminants of Emerging Concern (CECs): Distribution, Risks, and Implications for Water Quality and Health. Water.

[3] do Vale G.T., Ceron C.S., Gonzaga N.A., Simplicio J.A., Padovan J.C. (2018). Three Generations of β-Blockers: History, Class Differences and Clinical Applicability. Curr. Hypertens. Rev..

[4] U.S. Food and Drug Administration High Blood Pressure from the FDA Office of Women’s Health. https://www.fda.gov/consumers/womens-health-topics/high-blood-pressure#Beta_Blockers.

[5] Yan Y., An W., Mei S., Zhu Q., Li C., Yang L., Zhao Z., Huo J. (2024). Real-World Research on Beta-Blocker Usage Trends in China and Safety Exploration Based on the FDA Adverse Event Reporting System (FAERS). BMC Pharmacol. Toxicol..

[6] Gueye C., Cissé L., Diaw P.A., Mbaye O., Seye M. (2025). Analysis of Beta-Blockers in Environment—A Review. JSM Env. Sci. Ecol..

[7] Averbuch T., Esfahani M., Khatib R., Kayima J., Miranda J.J., Wadhera R.K., Zannad F., Pandey A., Van Spall H.G.C. (2023). Pharmaco-Disparities in Heart Failure: A Survey of the Affordability of Guideline Recommended Therapy in 10 Countries. ESC Heart Fail..

[8] Oliver E., Mayor F., D’Ocon P. (2019). Role of Beta-Blockers in Cardiovascular Disease in 2019. Rev. Española Cardiol..

[9] de Lucia C., Eguchi A., Koch W.J. (2018). New Insights in Cardiac β-Adrenergic Signaling during Heart Failure and Aging. Front. Pharmacol..

[10] Reiter M.J. (2004). Cardiovascular Drug Class Specificity: β-Blockers. Prog. Cardiovasc. Dis..

[11] Maszkowska J., Stolte S., Kumirska J., Łukaszewicz P., Mioduszewska K., Puckowski A., Caban M., Wagil M., Stepnowski P., Białk-Bielińska A. (2014). Beta-Blockers in the Environment: Part I. Mobility and Hydrolysis Study. Sci. Total Environ..

[12] Ulvi A., Aydın S., Aydın M.E. (2022). Fate of Selected Pharmaceuticals in Hospital and Municipal Wastewater Effluent: Occurrence, Removal, and Environmental Risk Assessment. Environ. Sci. Pollut. Res..

[13] Kasprzyk-Hordern B., Dinsdale R.M., Guwy A.J. (2009). The Removal of Pharmaceuticals, Personal Care Products, Endocrine Disruptors and Illicit Drugs during Wastewater Treatment and Its Impact on the Quality of Receiving Waters. Water Res..

[14] Xu J., Sun H., Zhang Y., Alder A.C. (2019). Occurrence and Enantiomer Profiles of β-Blockers in Wastewater and a Receiving Water Body and Adjacent Soil in Tianjin, China. Sci. Total Environ..

[15] Scheurer M., Ramil M., Metcalfe C.D., Groh S., Ternes T.A. (2010). The Challenge of Analyzing Beta-Blocker Drugs in Sludge and Wastewater. Anal. Bioanal. Chem..

[16] Biel-Maeso M., Corada-Fernández C., Lara-Martín P.A. (2018). Monitoring the Occurrence of Pharmaceuticals in Soils Irrigated with Reclaimed Wastewater. Environ. Pollut..

[17] Roberts J., Kumar A., Du J., Hepplewhite C., Ellis D.J., Christy A.G., Beavis S.G. (2016). Pharmaceuticals and Personal Care Products (PPCPs) in Australia’s Largest Inland Sewage Treatment Plant, and Its Contribution to a Major Australian River during High and Low Flow. Sci. Total Environ..

[18] Subedi B., Kannan K. (2015). Occurrence and Fate of Select Psychoactive Pharmaceuticals and Antihypertensives in Two Wastewater Treatment Plants in New York State, USA. Sci. Total Environ..

[19] Iancu V.I., Radu G.L., Scutariu R. (2019). A New Analytical Method for the Determination of Beta-Blockers and One Metabolite in the Influents and Effluents of Three Urban Wastewater Treatment Plants. Anal. Methods.

[20] Paíga P., Correia M., Fernandes M.J., Silva A., Carvalho M., Vieira J., Jorge S., Silva J.G., Freire C., Delerue-Matos C. (2019). Assessment of 83 Pharmaceuticals in WWTP Influent and Effluent Samples by UHPLC-MS/MS: Hourly Variation. Sci. Total Environ..

[21] Shao X.T., Zhao Y.T., Jiang B., Li Y.Y., Lin J.G., Wang D.G. (2023). Evaluation of Three Chronic Diseases by Selected Biomarkers in Wastewater. ACS ES T Water.

[22] Archer E., Petrie B., Kasprzyk-Hordern B., Wolfaardt G.M. (2017). The Fate of Pharmaceuticals and Personal Care Products (PPCPs), Endocrine Disrupting Contaminants (EDCs), Metabolites and Illicit Drugs in a WWTW and Environmental Waters. Chemosphere.

[23] Elorriaga Y., Marino D.J., Carriquiriborde P., Ronco A.E. (2013). Human Pharmaceuticals in Wastewaters from Urbanized Areas of Argentina. Bull. Env. Contam. Toxicol..

[24] Moslah B., Hapeshi E., Jrad A., Fatta-Kassinos D., Hedhili A. (2018). Pharmaceuticals and Illicit Drugs in Wastewater Samples in North-Eastern Tunisia. Environ. Sci. Pollut. Res..

[25] Verlicchi P., Al Aukidy M., Galletti A., Petrovic M., Barceló D. (2012). Hospital Effluent: Investigation of the Concentrations and Distribution of Pharmaceuticals and Environmental Risk Assessment. Sci. Total Environ..

[26] Oliveira T.S., Murphy M., Mendola N., Wong V., Carlson D., Waring L. (2015). Characterization of Pharmaceuticals and Personal Care Products in Hospital Effluent and Waste Water Influent/Effluent by Direct-Injection LC-MS-MS. Sci. Total Environ..

[27] Yu T.H., Lin A.Y.C., Wang X.H., Lin C.F. (2011). Occurrence of β-Blockers and β-Agonists in Hospital Effluents and Their Receiving Rivers in Southern Taiwan. Desalination Water Treat..

[28] Al Qarni H., Collier P., O’Keeffe J., Akunna J. (2016). Investigating the Removal of Some Pharmaceutical Compounds in Hospital Wastewater Treatment Plants Operating in Saudi Arabia. Environ. Sci. Pollut. Res..

[29] Wang L.S., Aziz Z., Wang E.S., Chik Z. (2024). Unused Medicine Take-Back Programmes: A Systematic Review. J. Pharm. Policy Pract..

[30] The OECD (Organisation for Economic Co-operation and Development) (2022). Management of Pharmaceutical Household Waste.

[31] Shaaban H., Alghamdi H., Alhamed N., Alziadi A., Mostafa A. (2018). Environmental Contamination by Pharmaceutical Waste: Assessing Patterns of Disposing Unwanted Medications and Investigating the Factors Influencing Personal Disposal Choices. J. Pharmacol. Pharm. Res..

[32] Vulliet E., Cren-Olivé C., Grenier-Loustalot M.F. (2011). Occurrence of Pharmaceuticals and Hormones in Drinking Water Treated from Surface Waters. Env. Chem. Lett..

[33] Ma R., Qu H., Wang B., Wang F., Yu G. (2020). Widespread Monitoring of Chiral Pharmaceuticals in Urban Rivers Reveals Stereospecific Occurrence and Transformation. Env. Int..

[34] Yao B., Yan S., Lian L., Yang X., Wan C., Dong H., Song W. (2018). Occurrence and Indicators of Pharmaceuticals in Chinese Streams: A Nationwide Study. Environ. Pollut..

[35] Giebułtowicz J., Stankiewicz A., Wroczyński P., Nałęcz-Jawecki G. (2016). Occurrence of Cardiovascular Drugs in the Sewage-Impacted Vistula River and in Tap Water in the Warsaw Region (Poland). Environ. Sci. Pollut. Res..

[36] Huerta-Fontela M., Galceran M.T., Ventura F. (2011). Occurrence and Removal of Pharmaceuticals and Hormones through Drinking Water Treatment. Water Res..

[37] Varga R., Somogyvári I., Eke Z., Torkos K. (2013). Seasonal Monitoring of Cardiovascular and Antiulcer Agents’ Concentrations in Stream Waters Encompassing a Capital City. J. Pharm..

[38] Nantaba F., Wasswa J., Kylin H., Palm W.U., Bouwman H., Kümmerer K. (2020). Occurrence, Distribution, and Ecotoxicological Risk Assessment of Selected Pharmaceutical Compounds in Water from Lake Victoria, Uganda. Chemosphere.

[39] Sodré F.F., Santana J.S., Sampaio T.R., Brandão C.C.S. (2018). Seasonal and Spatial Distribution of Caffeine, Atrazine, Atenolol and Deet in Surface and Drinking Waters from the Brazilian Federal District. J. Braz. Chem. Soc..

[40] Rivera-Jaimes J.A., Postigo C., Melgoza-Alemán R.M., Aceña J., Barceló D., López de Alda M. (2018). Study of Pharmaceuticals in Surface and Wastewater from Cuernavaca, Morelos, Mexico: Occurrence and Environmental Risk Assessment. Sci. Total Environ..

[41] Nödler K., Hillebrand O., Idzik K., Strathmann M., Schiperski F., Zirlewagen J., Licha T. (2013). Occurrence and Fate of the Angiotensin II Receptor Antagonist Transformation Product Valsartan Acid in the Water Cycle—A Comparative Study with Selected β-Blockers and the Persistent Anthropogenic Wastewater Indicators Carbamazepine and Acesulfame. Water Res..

[42] Gworek B., Kijeńska M., Wrzosek J., Graniewska M. (2021). Pharmaceuticals in the Soil and Plant Environment: A Review. Water Air Soil Pollut..

[43] Grossberger A., Hadar Y., Borch T., Chefetz B. (2014). Biodegradability of Pharmaceutical Compounds in Agricultural Soils Irrigated with Treated Wastewater. Environ. Pollut..

[44] Aznar R., Sánchez-Brunete C., Albero B., Rodríguez J.A., Tadeo J.L. (2014). Occurrence and Analysis of Selected Pharmaceutical Compounds in Soil from Spanish Agricultural Fields. Environ. Sci. Pollut. Res..

[45] Siebert C.D., Hänsicke A., Nagel T. (2008). Stereochemical Comparison of Nebivolol with Other β-Blockers. Chirality.

[46] Ribeiro A.R., Castro P.M.L., Tiritan M.E. (2012). Chiral Pharmaceuticals in the Environment. Env. Chem. Lett..

[47] Kasprzyk-Hordern B. (2010). Pharmacologically Active Compounds in the Environment and Their Chirality. Chem. Soc. Rev..

[48] Hernando M.D., Gómez M.J., Agüera A., Fernández-Alba A.R. (2007). LC-MS Analysis of Basic Pharmaceuticals (Beta-Blockers and Anti-Ulcer Agents) in Wastewater and Surface Water. TrAC Trends Anal. Chem..

[49] Hanley M.J., Abernethy D.R., Greenblatt D.J. (2010). Effect of Obesity on the Pharmacokinetics of Drugs in Humans. Clin. Pharmacokinet..

[50] Ramil M., El Aref T., Fink G., Scheurer M., Ternes T.A. (2010). Fate of Beta Blockers in Aquatic-Sediment Systems: Sorption and Biotransformation. Env. Sci. Technol..

[51] Yi M., Sheng Q., Sui Q., Lu H. (2020). β-Blockers in the Environment: Distribution, Transformation, and Ecotoxicity. Environ. Pollut..

[52] Barbato F., Di Martino G., Grumetto L., La Rotonda M.I. (2005). Can Protonated β-Blockers Interact with Biomembranes Stronger than Neutral Isolipophilic Compounds? A Chromatographic Study on Three Different Phospholipid Stationary Phases (IAM-HPLC). Eur. J. Pharm. Sci..

[53] Pereira-Leite C., Carneiro C., Soares J.X., Afonso C., Nunes C., Lúcio M., Reis S. (2013). Biophysical Characterization of the Drug-Membrane Interactions: The Case of Propranolol and Acebutolol. Eur. J. Pharm. Biopharm..

[54] Kibbey T.C.G., Paruchuri R., Sabatini D.A., Chen L. (2007). Adsorption of Beta Blockers to Environmental Surfaces. Env. Sci. Technol..

[55] Schaffer M., Börnick H., Nödler K., Licha T., Worch E. (2012). Role of Cation Exchange Processes on the Sorption Influenced Transport of Cationic β-Blockers in Aquifer Sediments. Water Res..

[56] Smith R.M., Sayen S., Guillon E. (2022). Adsorption of Individual and Mixtures of β-Blockers and Copper in Soils and Sediments. Env. Toxicol. Chem..

[57] Ambrozini B., Cervini P., Cavalheiro É.T.G. (2016). Thermal Behavior of the β-Blocker Propranolol. J. Therm. Anal. Calorim..

[58] Krzek J., Kwiecień A., Zylewski M. (2006). Stability of Atenolol, Acebutolol and Propranolol in Acidic Environment Depending on Its Diversified Polarity. Pharm. Dev. Technol..

[59] Peikova L., Pencheva I., Tzvetkova B. (2013). Chemical Stability-Indicating HPLC Study of Fixed-Dosage Combination Containing Metoprolol Tartrate and Hydrochlorothiazide. J. Chem. Pharm. Res..

[60] Kovácsa K., Tóth T., Wojnárovits L. (2022). Evaluation of Advanced Oxidation Processes for β-Blockers Degradation: A Review. Water Sci. Technol..

[61] Piram A., Salvador A., Verne C., Herbreteau B., Faure R. (2008). Photolysis of β-Blockers in Environmental Waters. Chemosphere.

[62] Liu Q.T., Williams H.E. (2007). Kinetics and Degradation Products for Direct Photolysis of β-Blockers in Water. Env. Sci. Technol..

[63] Makunina M.P., Pozdnyakov I.P., Chen Y., Grivin V.P., Bazhin N.M., Plyusnin V.F. (2015). Mechanistic Study of Fulvic Acid Assisted Propranolol Photodegradation in Aqueous Solution. Chemosphere.

[64] Chmiel T., Mieszkowska A., Kempińska-Kupczyk D., Kot-Wasik A., Namieśnik J., Mazerska Z. (2019). The Impact of Lipophilicity on Environmental Processes, Drug Delivery and Bioavailability of Food Components. Microchem. J..

[65] Liu J., Lu G., Xie Z., Zhang Z., Li S., Yan Z. (2015). Occurrence, Bioaccumulation and Risk Assessment of Lipophilic Pharmaceutically Active Compounds in the Downstream Rivers of Sewage Treatment Plants. Sci. Total Environ..

[66] Salmina E.S., Wondrousch D., Kühne R., Potemkin V.A., Schüürmann G. (2016). Variation in Predicted Internal Concentrations in Relation to PBPK Model Complexity for Rainbow Trout. Sci. Total Environ..

[67] Lin W., Huang Z., Ping S., Zhang S., Wen X., He Y., Ren Y. (2022). Toxicological Effects of Atenolol and Venlafaxine on Zebrafish Tissues: Bioaccumulation, DNA Hypomethylation, and Molecular Mechanism. Environ. Pollut..

[68] Rojo M., Cristos D., González P., López-Aca V., Dománico A., Carriquiriborde P. (2021). Accumulation of Human Pharmaceuticals and Activity of Biotransformation Enzymes in Fish from Two Areas of the Lower Rio de La Plata Basin. Chemosphere.

[69] Ruan Y., Lin H., Zhang X., Wu R., Zhang K., Leung K.M.Y., Lam J.C.W., Lam P.K.S. (2020). Enantiomer-Specific Bioaccumulation and Distribution of Chiral Pharmaceuticals in a Subtropical Marine Food Web. J. Hazard. Mater..

[70] Rutere C., Posselt M., Ho A., Horn M.A. (2021). Biodegradation of Metoprolol in Oxic and Anoxic Hyporheic Zone Sediments: Unexpected Effects on Microbial Communities. Appl. Microbiol. Biotechnol..

[71] Kodešová R., Klement A., Golovko O., Fér M., Nikodem A., Kočárek M., Grabic R. (2019). Root Uptake of Atenolol, Sulfamethoxazole and Carbamazepine, and Their Transformation in Three Soils and Four Plants. Environ. Sci. Pollut. Res..

[72] Yi M., Sheng Q., Lv Z., Lu H. (2022). Novel Pathway and Acetate-Facilitated Complete Atenolol Degradation by Hydrogenophaga Sp. YM1 Isolated from Activated Sludge. Sci. Total Environ..

[73] Yi M., Lou J., Zhu W., Li D., Yu P., Lu H. (2023). Mechanism of β-Blocker Biodegradation by Wastewater Microorganisms. J. Hazard. Mater..

[74] Xu Y., Radjenovic J., Yuan Z., Ni B.J. (2017). Biodegradation of Atenolol by an Enriched Nitrifying Sludge: Products and Pathways. Chem. Eng. J..

[75] Rezaei R., Aghapour A.A., Khorsandi H. (2022). Investigating the Biological Degradation of the Drug β-Blocker Atenolol from Wastewater Using the SBR. Biodegradation.

[76] Zhou J., Wang D., Ju F., Hu W., Liang J., Bai Y., Liu H., Qu J. (2022). Profiling Microbial Removal of Micropollutants in Sand Filters: Biotransformation Pathways and Associated Bacteria. J. Hazard. Mater..

[77] Koba O., Golovko O., Kodešová R., Klement A., Grabic R. (2016). Transformation of Atenolol, Metoprolol, and Carbamazepine in Soils: The Identification, Quantification, and Stability of the Transformation Products and Further Implications for the Environment. Environ. Pollut..

[78] Rubirola A., Llorca M., Rodriguez-Mozaz S., Casas N., Rodriguez-Roda I., Barceló D., Buttiglieri G. (2014). Characterization of Metoprolol Biodegradation and Its Transformation Products Generated in Activated Sludge Batch Experiments and in Full Scale WWTPs. Water Res..

[79] Jaén-Gil A., Castellet-Rovira F., Llorca M., Villagrasa M., Sarrà M., Rodríguez-Mozaz S., Barceló D. (2019). Fungal Treatment of Metoprolol and Its Recalcitrant Metabolite Metoprolol Acid in Hospital Wastewater: Biotransformation, Sorption and Ecotoxicological Impact. Water Res..

[80] Maurer M., Escher B.I., Richle P., Schaffner C., Alder A.C. (2007). Elimination of β-Blockers in Sewage Treatment Plants. Water Res..

[81] Ribeiro A.R., Afonso C.M., Castro P.M.L., Tiritan M.E. (2013). Enantioselective Biodegradation of Pharmaceuticals, Alprenolol and Propranolol, by an Activated Sludge Inoculum. Ecotoxicol. Env. Saf..

[82] Tang Y., Zhao B., Liu C. (2020). Removal Mechanisms of β-Blockers by Anaerobic Digestion in a UASB Reactor with Carbon Feeding. Bioresour. Technol. Rep..

[83] Triebskorn R., Casper H., Scheil V., Schwaiger J. (2007). Ultrastructural Effects of Pharmaceuticals (Carbamazepine, Clofibric Acid, Metoprolol, Diclofenac) in Rainbow Trout (*Oncorhynchus mykiss*) and Common Carp (*Cyprinus carpio*). Anal. Bioanal. Chem..

[84] Maranho L.A., André C., DelValls T.A., Gagné F., Martín-Díaz M.L. (2015). Toxicological Evaluation of Sediment Samples Spiked with Human Pharmaceutical Products: Energy Status and Neuroendocrine Effects in Marine Polychaetes Hediste Diversicolor. Ecotoxicol. Env. Saf..

[85] Jeong T.Y., Yoon D., Kim S., Kim H.Y., Kim S.D. (2018). Mode of Action Characterization for Adverse Effect of Propranolol in Daphnia Magna Based on Behavior and Physiology Monitoring and Metabolite Profiling. Environ. Pollut..

[86] Parrott J.L., Balakrishnan V.K. (2017). Life-Cycle Exposure of Fathead Minnows to Environmentally Relevant Concentrations of the Β-Blocker Drug Propranolol. Env. Toxicol. Chem..

[87] Feiner M., Laforsch C., Letzel T., Geist J. (2014). Sublethal Effects of the Beta-Blocker Sotalol at Environmentally Relevant Concentrations on the New Zealand Mudsnail Potamopyrgus Antipodarum. Env. Toxicol. Chem..

[88] Salinas A. (2024). Effects of Metoprolol and Propranolol Beta-Blockers Mixture on Effects of Metoprolol and Propranolol Beta-Blockers Mixture on Morphological and Physiological Responses in the American Morphological and Physiological Responses in the American Oyster Oyster. Master’s Thesis.

[89] Godoy A.A., Domingues I., de Carvalho L.B., Oliveira Á.C., de Jesus Azevedo C.C., Taparo J.M., Assano P.K., Mori V., de Almeida Vergara Hidalgo V., Nogueira A.J.A. (2020). Assessment of the Ecotoxicity of the Pharmaceuticals Bisoprolol, Sotalol, and Ranitidine Using Standard and Behavioral Endpoints. Environ. Sci. Pollut. Res..

[90] Liu Q.-T., Williams T.D., Cumming R.I., Holm G., Hetheridge M.J. (2009). Comparative Aquatic Toxicity of Propranolol and Its Photodegraded Mixtures: Algae and Rotifer Screening. Env. Toxicol. Chem..

[91] Maszkowska J., Stolte S., Kumirska J., Łukaszewicz P., Mioduszewska K., Puckowski A., Caban M., Wagil M., Stepnowski P., Białk-Bielińska A. (2014). Beta-Blockers in the Environment: Part II. Ecotoxicity Study. Sci. Total Environ..

[92] Duarte B., Feijão E., de Carvalho R.C., Duarte I.A., Silva M., Matos A.R., Cabrita M.T., Novais S.C., Lemos M.F.L., Marques J.C. (2020). Effects of Propranolol on Growth, Lipids and Energy Metabolism and Oxidative Stress Response of Phaeodactylum Tricornutum. Biology.

[93] Finn J., Hui M., Li V., Lorenzi V., de la Paz N., Cheng S.H., Lai-Chan L., Schlenk D. (2012). Effects of Propranolol on Heart Rate and Development in Japanese Medaka (Oryzias Latipes) and Zebrafish (Danio Rerio). Aquat. Toxicol..

[94] Margiotta-Casaluci L., Owen S.F., Rand-Weaver M., Winter M.J. (2019). Testing the Translational Power of the Zebrafish: An Inter-Species Analysis of Responses to Cardiovascular Drugs. Front. Pharmacol..

[95] Mitchell K.M., Moon T.W. (2016). Behavioral and Biochemical Adjustments of the Zebrafish Danio Rerio Exposed to the β-Blocker Propranolol. Comp. Biochem. Physiol. B Biochem. Mol. Biol..

[96] Cheong H.I., Johnson J., Cormier M., Hosseini K. (2008). In Vitro Cytotoxicity of Eight β-Blockers in Human Corneal Epithelial and Retinal Pigment Epithelial Cell Lines: Comparison with Epidermal Keratinocytes and Dermal Fibroblasts. Toxicol. Vitr..

[97] Četojević-Simin D.D., Armaković S.J., Šojić D.V., Abramović B.F. (2013). Toxicity Assessment of Metoprolol and Its Photodegradation Mixtures Obtained by Using Different Type of TiO2 Catalysts in the Mammalian Cell Lines. Sci. Total Environ..

[98] Oskarsson H., Wiklund A.K.E., Thorsén G., Danielsson G., Kumblad L. (2014). Community Interactions Modify the Effects of Pharmaceutical Exposure: A Microcosm Study on Responses to Propranolol in Baltic Sea Coastal Organisms. PLoS ONE.

[99] Lindim C., van Gils J., Georgieva D., Mekenyan O., Cousins I.T. (2016). Evaluation of Human Pharmaceutical Emissions and Concentrations in Swedish River Basins. Sci. Total Environ..

[100] Sarabyar S., Farahbakhsh A., Tahmasebi H.A., Mahmoodzadeh Vaziri B., Khosroyar S. (2024). Enhancing Photocatalytic Degradation of Beta-Blocker Drugs Using TiO2 NPs/Zeolite and ZnO NPs/Zeolite as Photocatalysts: Optimization and Kinetic Investigations. Sci. Rep..

